# ANN-ANFIS model for optimising methylic composite biodiesel from neem and castor oil and predicting emissions of the biodiesel blend

**DOI:** 10.1038/s41598-025-88901-9

**Published:** 2025-02-15

**Authors:** Chao-zhe Zhu, Olusegun David Samuel, Amin Taheri-Garavand, Noureddine Elboughdiri, Prabhu Paramasivam, Fayaz Hussain, Christopher C. Enweremadu, Abinet Gosaye Ayanie

**Affiliations:** 1 School of Medical Engineering, Jining Medical University, Jining City, Shandong Province China; 2 Department of Mechanical Engineering, Federal University of Petroleum Resources, P.M.B 1221, Effurun, Delta State Nigeria; 3 Mechanical Engineering of Biosystems Department, Lorestan University, Khorramabad, Iran; 4 Chemical Engineering Department, College of Engineering, University of Ha’il, P.O. Box 2440, Ha’il, 81441 Saudi Arabia; 5 Chemical Engineering Process Department, National School of Engineers Gabes, University of Gabes, Gabes, 6029 Tunisia; 6 Department of Research and Innovation, Saveetha School of Engineering, SIMATS, Chennai, Tamilnadu 602105 India; 7 Department of Biological and Agricultural Engineering, Faculty of Engineering, Universiti Putra Malaysia, Selangor, Malaysia; 8 Department of Mechanical, Bioresources and Biomedical Engineering, Science Campus, University of South Africa, Private Bag X6, Florida, 1709 South Africa; 9 Department of Mechanical Engineering, Adama Science and Technology University, Adama, 2552 Ethiopia

**Keywords:** Composite biodiesel, ANN, ANFIS, Emission characteristics, Optimization, Mechanical engineering, Nanoscience and technology

## Abstract

Researchers and stakeholders have shown interest in heterogeneous composite biodiesel (HCB) due to its enhanced fuel properties and environmental friendliness (EF). The lack of high viscosity datasets for parent hybrid oils has hindered their commercialisation. Reliable models are lacking to optimise the transesterification parameters for developing HCB, and the scarcity of predictive models has affected climate researchers and environmental experts. In this study, basic fuel properties were analysed, and models were developed models for the yield of HCB and kinematic viscosity (KV) for composite biodiesel/neem castor seed oil methyl ester (NCSOME) using Artificial Neural Network (ANN) and Adaptive Neuro Fuzzy Inference System (ANFIS). Statistical indices such as computed coefficient of determination (R^2^), root-mean-square-error (RMSE), standard error of prediction (SEP), mean average error (MAE), and average absolute deviation (AAD) were used to evaluate the effectiveness of the techniques. Emission models for NCSOME-diesel blends were also established. The study investigated the impact of optimised fuel types/NCSOME-diesel (10–30 vol%), ZnO nanoparticle dosage (400–800 ppm), engine speed (1100–1700 rpm), and engine load (10–30%) on emission characteristics and environmental friendliness indices (EFI) such as carbon monoxide (CO), Oxides of Nitrogen (NOx), and Unburnt Hydrocarbon (UHC) using Response Surface Methodology (RSM). The ANFIS model demonstrated superior performance in terms of R^2^, RMSE, SEP, MAE, and AAD compared to the ANN model in predicting yield and KV of HCB. The optimal emission levels for CO (49.26 ppm), NO_x_ (0.5171 ppm), and UHC (2.783) were achieved with a fuel type of 23.4%, nanoparticle dosage of 685.432 ppm, engine speed of 1329.2 rpm, and engine load of 10% to ensure cleaner EFI. The hybrid ANFIS and ANN models can effectively predict and model fuel-related characteristics and improve the HCB process, while the RSM model can be a valuable tool for climate and environmental stakeholders in accurate forecasting and promoting a cleaner environment. The valuable datasets can also provide reliable information for strategic planning in the biodiesel and automotive industries.

**Supplementary Information:**

The online version contains supplementary material available at 10.1038/s41598-025-88901-9.

## Introduction

The fact that fossil fuels meet most of the world’s energy needs shows that people are heavily dependent on non-renewable resources^1,2^. Global energy consumption is expected to increase by 56% between 2010 and 2040. In addition to limited resources, the use of fossil fuels as the main energy source brings with it many concerns about the environment and health. This situation shows that there is an urgent need for energy-efficient products. Biofuels have gained international recognition due to their environmentally beneficial, clean, sustainable and biodegradable qualities^3–5^.

In the face of the global energy crisis, environmental pollution, and health risks associated with oil-based products, it is imperative to explore alternative fuels. Osman et al.^6^ underscored the importance of implementing policies that promote the efficient use of waste resources and reduce greenhouse gas emissions for a sustainable future.

Biodiesel is a sustainable energy source with some similarities to petrochemical diesel, such as a high cetane number, high flash point and high combustion efficiency^7^. Biodiesel can be produced from vegetable or animal oils and is a green energy source because it is environmentally friendly^8^. Biodiesel made from blended fuels has special fuel properties that can increase fuel efficiency and engine power when used in an internal combustion engine. Additionally, biodiesel produced from oil blending not only eliminates food-oil incompatibility but also enhances the stability of biodiesel and reduces production costs. There are various technologies for producing biodiesel, but the most important one is transesterification. The popularity of transesterification in biodiesel production is due to its superior quality^9^.

The selection of suitable raw materials that are cheap, locally available and non-toxic is important for the commercialisation of biodiesel production^10^. In addition, hybrid oils such as castor oil and neem seed, which are often referred to as hybrid second-generation raw materials, are often underused in tropical regions and around the world and are not suitable for human consumption. Both homogeneous and heterogeneous catalysts (HOC and HEC, respectively) can be used in the transesterification reaction; however, heterogeneous acid catalysts are preferred due to their ability to withstand fatty acids found in crude oil and have advantages such as easier separation and reusability^11^. Among the bioorganic wastes generated for HEC, eggshells and animal bones stand out with their innovative nature, green properties of catalysts and ability to prevent environmental and economic damages^12^.

The production of biomass diesel by HC-catalyzed transesterification reaction has attracted researchers and stakeholders. Most of the research in this area aims to increase the efficiency of biodiesel production by testing the process, developing new catalysts, and optimizing the process^13^. Although the transesterification process plays an important role in many hybrid feedback and reaction systems (MI-HR), it is important to optimise the MI-HR to achieve the desired successful reaction smoothly. The use of artificial intelligence (AI) to model the biodiesel production process and reduce the kinematic viscosity (KV) has become popular. Optimisation and evaluation of biodiesel yield and KV using AI require small-scale and efficient experiments.

Adaptive neuro-fuzzy inference systems (ANFIS) and artificial neural networks (ANN) are branches of artificial intelligence focused on efficient control procedures, task classification, task prediction, and problem-solving^14^. ANFIS is an artificial intelligence system that simulates human reasoning to solve problems. In ANFIS, the input and output information of the engineering process are represented as IF-THEN fuzzy rules. This allows for the combination of neural network learning with fuzzy logic to better represent thinking. On the other hand, ANNs are computational models useful for prediction and classification tasks in data processing. They are inspired by the information processing characteristics of organisms such as the human brain to acquire knowledge and use it for prediction and classification in data processing^15^. ANNs can predict outcomes by adjusting the weights and biases (learning) in the network to capture linear and nonlinear patterns in data while maintaining constraints^16^.

ANFIS and ANN have been used in many biodiesel studies, such as prediction based on fatty acids^17^, prediction of biodiesel from supercritical fuels growth^18^, biodiesel dynamic viscosity prediction^19^, biodiesel cooling flow prediction^20^, reactor temperature control^21^, water content of biodiesel/diesel blends^22^, ANFIS based mechanical and ultrasonic mixing^23^, optimization of rice bran oil^24^, density, viscosity and cetane number model^25^. Beticu et al.^26^ and Agu et al.^27^ reported that ANFIS has a higher capacity than the ANN technique. However, these techniques can be used to build reliable models for accurate prediction in various tasks such as compressive strength of cement-based mortar data^28^, ground platform prediction in Iran^29^, and flood flow prediction^30^. Recently, Deka et al.^31^ utilized the Partial Reinforcement Optimiser (PRO) algorithm integrated with Random Vector Functional Link (RVFL) to improve engine performance estimation for a diesel engine running on methanol/diesel blends. Their findings suggest that the novel PRO-RVFL models support the advancement of cleaner and more efficient transportation systems.

The environmental friendliness of biodiesel adoption in diesel engines depends on its lower pollutant emissions^32^. Running diesel engines on fossil diesel leads to increased nitrogen oxide emissions, rising fossil diesel prices, and a significant decline in reserves^33^. Faruque et al.^34^ and Kataria et al.^35^ suggested that heterogeneous catalyzed composite or mono biodiesels doped with nanoparticles are able to overcome these challenges. To evaluate the environmental friendliness of green diesel, emission features such as carbon dioxide, carbon monoxide, and exhaust smoke are examined^36^. The emission characteristics of IC engines running on the aforementioned biodiesels doped with nanoparticles are highlighted in Table 2.

Composite biodiesel blends from multiple oils have synergistic fuel properties that improve diesel engine performance, but they often do not reduce emissions. Adding nanoparticles to composite biodiesel can help reduce unwanted emissions^37^. The oxygenated nature of nanoparticles enhances emission reduction. Various types of nanoparticles have been found to improve emission characteristics^38^. Commonly used nanoparticles include cerium dioxide, aluminum oxide, titanium oxide, copper oxide, zinc oxide, iron oxide, and carbon nanotubes^39–42^. Among these nanoparticles, ZnO is preferred for its ability to enhance micro-explosion occurrence, increase oxygen levels, boost ignition velocity, and improve fuel combustion^43,44^. To the best of our knowledge, the environmental friendliness of heterogeneous composite biodiesel from neem-castor biodiesel doped with ZnO exhaust from IC engines has not been studied.

### Optimal emission reduction prediction for environmental sustainability

Predicting optimal emission reductions is crucial for environmental sustainability as Emission Features (EFs) directly impact environmental indicators. Natrayan et al.^45^ emphasised the significance of predicting optimal emission reductions for environmental sustainability, as EFs directly affect environmental indicators. EFs such as CO_2_, NO_x_, and unburnt hydrocarbons play a crucial role in climate action aligned with the UN Sustainable Development Goals 2020^46,47^. Precise forecasting of EFs is vital for achieving a sustainable environment^48^. The use of RSM can establish multi-response models for effective forecasting and modelling between engine inputs and IC engines powered by low carbon fuel/diesel doped with nanoparticles to reduce EFs^49^. This approach helps combat climate change, enhance air quality, and protect ecosystems. Accurate predictions assist policymakers and businesses in making informed decisions on emission reduction strategies, setting attainable targets, and monitoring progress towards sustainability goals^50^. This study aims to offer practical emission reduction strategies and reliable models for policy makers and stakeholders, applicable in real-world scenarios, and contributing to environmental sustainability.

### Motivation, novelty and aim of the study

Although there are many artificial intelligence (AI) models in engineering, the combination of ANNs and ANFIS models remains important due to the ANFIS model’s capability to solve constraints and handle nonlinear data. The ANFIS model does not suffer from these limitations^51^. Moreover, the integration of ANN-ANFIS models can handle nonlinear models, complex stochastic data, complex operations and produce good results^52^. However, deep learning (DL) models are often used to address the limitations of soft computing models (SCM) like ANN, and ANFIS. However, a small dataset can lead to overfitting in DL models, where the model performs well on training data but poorly on new testing data, reducing generalisability and transferability^53^.The data requirements of DL make it challenging to obtain precise results, hindering its adoption with small datasets, while SCM can effectively model datasets of various sizes.

In the context of biodiesel production and emission modelling, the datasets used are of medium size. Due to DL’s struggles with optimising biodiesel modelling and emission prediction with medium datasets, the authors opted for SCM to establish reliable and accurate predictions and models.

Table 1 provides an overview of the applications of various AI techniques in green diesel production. To the best of our knowledge, a hybrid tool combining ANN and ANFIS has not been explored for modelling transesterification parameters in the production of composite biodiesel from non-conventional seed oils (NCSO) using eggshell and animal bone waste as a heterogeneous catalyst. Research on the use of bio-agricultural waste as a catalyst for the methylation process of NCSO and the kinematic study of production can help facilitate its adoption in IC engines. However, a comprehensive review of current research and AI-based analysis of composite biodiesel using a heterogeneous catalyst revealed several gaps: (1) the absence of an established ANN model for NCSOME production, (2) the lack of ANFIS-based prediction for bio-waste catalyzed transesterification of oils from castor and neem seeds, (3) limited comparative studies on hybrid models for composite biodiesel parameters, and (4) the necessity for predictive models to reduce emission profiles using heterogeneous biodiesel doped with ZnO nanoparticles. These gaps have prompted further investigation to develop ANN and ANFIS techniques for NCSOME production using a novel bio-waste catalyst, compare the effectiveness of ANFIS and ANN models, and establish regression models for exhaust emission profiles in engines fuelled by composite biodiesel.

**Table 1:** Comparative analysis of AI models in biodiesel production.

Types of feedstock oil(s)	Model tools	Nature of Catalyst	Types of BD: HB or NHB	Response(s)	Remarks	Technical deficiency	Refs.
Waste cooking oil, waste palm oil, and waste animal fat	ANN, SVM, and ELM	Waste marble tile and plantain peduncle	HB	Yield	The relevance of Ml showcased in HB production	Single response (yield) reported	Amenaghawon et al.^54^
Karanja oil	ANFIS, ANN, RSM	NaOH	NHB	Yield	Highest yield obtained by different model tools	Single oil & seed response (yield ) reported	Kumar et al.^55^
Waste cooking oil	Three machine learning (ML)-based approaches and GA		HB	Yield	Efficacy of the 3 ML tools reported	ML based for modelling mono-oily biodiesel	Ahmad et al.^56^
African pear seed	ANFIS and ANN models	Clay based HEC	NHB	Cetane number, KV	Accurate prediction of KV and CN using ANFIS and ANN models	Single oily feedstock adopted	Ude et al.^57^
Mixture of palm and cotton seed oil	RSM-ANN	KOH	HB	Yield	ANN outperformed RSM	The hybrid model is established for homogeneous based HB	Razzaq et al.^58^

HB = hybrid biodiesel, ANN = artificial neural network, HEC = heterogeneous catalyst.

The study aimed to address the research gap and improve methyl yield by: (i) using central composite rotatable design of RSM to determine optimal conditions for methanol/NCSO molar ratio (4–6), catalyst amount (12–20 wt%), reaction time (1–3 h), and temperature (50–70 ^o^C) on NCSOME yield and KV, (ii) defining optimal response variables using desirability approach of RSM, (iii) evaluating ANN and ANFIS as soft computing optimization tools for composite biodiesel production from pre-treated hybrid oils, (iv) analyzing NCSOME fuel characteristics under optimal conditions according to biodiesel standards, and (v) developing emission models for NCSOME doped with ZnO in fuel engines.

**Table 2:** Overview of key emission features of IC engines powered by biodiesels from diverse oils.

BD from oily feedstock	Model tool	Types of nanoparticles (NPs)	Emission features of IC engines	Remarks and knowledge in gaps	Refs.
Ceiba Penandra, Mahua Longifolia, and Azadirachta indica oil (CMAO)	DBN, AOA	CaO–TiO_2_	CO, NO_x_	Optimized Emission features responses for biodiesel from CMAO Models are not suitable for biodiesel from hybrid oils doped with ZnO	Sujin et al.^37^
Linseed oil (LO)	RSM and ANN	Graphene	CO, NO_x_, HC	Models for IC engine emission profile running on LOBD Models do not possess capacity to prediction for CB-NPs fueled engines	Rao et al.^59^
Waste coconut and fish oil	DoE and meta-heuristic algorithms	MgO	CO, NO_x_, UHC	Models derived empirical equations are applicable for prediction of emission features of IC operated on WC-FO BD doped with MGO Models were not established for the BD from hybrid oils and NPs currently studied	Dharmegowda et al.^60^
Pumpkin + Prosopis juliflora (PPJO)	RSM, MPS, DFA modified particle swarm and dragon fly algorithm combined with RSM technique	Nil	CO_2_, NO, HC	Models were not postulated for predicting NPs doped with CB	Viswanathan et al.^38^
Waste cooking oil biodiesel + Yang-hard resin	RSM	Nil	CO, CO_2_, and NO_X_	Optimal conditions for emission features established Emission models were only postulated for BD from mono oil	Katekaew et al.^61^
Roselle and Karanja oil (RKO)	OVAT	Nil	CO_2_, NO_x_, smoke	Feasibility of adopting CB from PKO for emission reduction documented Absence of emission models for predicting of emission tail profile fuellled RKO BD	Shrivastava et al.^62^
Jatropha and Alexandrian laurel (JALO)	OVAT	Nil	CO, HC, CO_2_, NOx	Major emission reduction stated for JALO BD application Nonappearance of emission models for forecasting of emission tail profile fuellled JALO BD	Ruhul et al.^63^
Palm-jatropha oil (PJO)	OVAT	Nil	NO	Major emission reduction reported for PJO BD utilization Nonappearance of emission models for forecasting of emission tail profile fuellled PJO BD	Sanjid et al.^64^

BD  = biodiesel; NOx  = oxides of Nitrogen, CO_2_ = carbon dioxide, UHC = unburnt hydrocarbon, CO = carbon monoxide; DBN = Deep belief network, AOA = Arithmetic optimization algorithm (AOA), MPS = modified particle swarm, DFA = dragon fly algorithm.

## Materials and methods

### NCSO analysis, heterogeneous catalyst and its metylic production

Neem oil was procured from Kano State, while castor was obtained from Oyo state, Nigeria. The ASTM standard was used to assess NCSO’s basic properties such as density, viscosity, acidity, and saponification value. Table 3 summarizes the equipment and methods applied, while Table 4 presents a list of equipment used.

**Table 3:** Major chemical constituents adopted.

Substance	Formula	Molecular weight (g/mol)	Specific gravity	Maker	Purity%
Methanaol	CH_3_OH	32.04	0.7917	JHD, China	
Ethanol	C_2_H_5_OH	46.07	0.789	JHD, China	99.6
Potassium hydroxide	NaOH	40	2.13	JHD, China	99.9
Sulphuric acid	H_2_SO_4_	98	1.84	Loba Chemie, India	98
Benzene	C_6_H_6_	78.11	0.876	BDH, England	99.5
Phenolphthalein	C_20_H_14_O_4_	318.32	1.28	Kermel, England	50

**Table 4:** List of apparatus.

Property	Equipment	Manufacturer
Viscosity	NDJ-5 S Viscometer and	Anton Paar, UK
Density	SVM 3000 50milliliter Pycnometer (Density Bottles)	Anton Paar, UK
Water bath	HJ-3D Constant Temperature Water bath	B. Brian, England
Magnetic stirrer	MS300 Constant temperature magnetic stirrer	Jinotech Sc. China
Drying	Vacutherm VT 6025 Vacuum Oven.	Thermo Sc. NJ, USA

The purpose was to produce methylic biodiesel from a mixture of neem and castor seed oil (NCSO) with methanol as the alcohol, and eggshells and animal bone as bio-waste for the production of a heterogeneous catalyst.

### Characterisation of heterogeneous catalysis and NCSOME production

The eggshell and cow bones were synthesied by calcining them in a temperature-controlled furnace at 800 ˚C for 3 h. The calcined catalyst powder was allowed to cool at room temperature in the furnace for 60 h before characterisation. The bio-waste heterogeneous catalyst (BWHC), namely eggshell-cow bones heterogeneous catalyst (ECHC), was characterised using Fourier transform infrared spectroscopy (FTIR) to confirm the presence of functional groups and verify the characteristic absorption bands of the catalysts as stipulated by Khan et al.^65,66^.

**Fig. 1:**
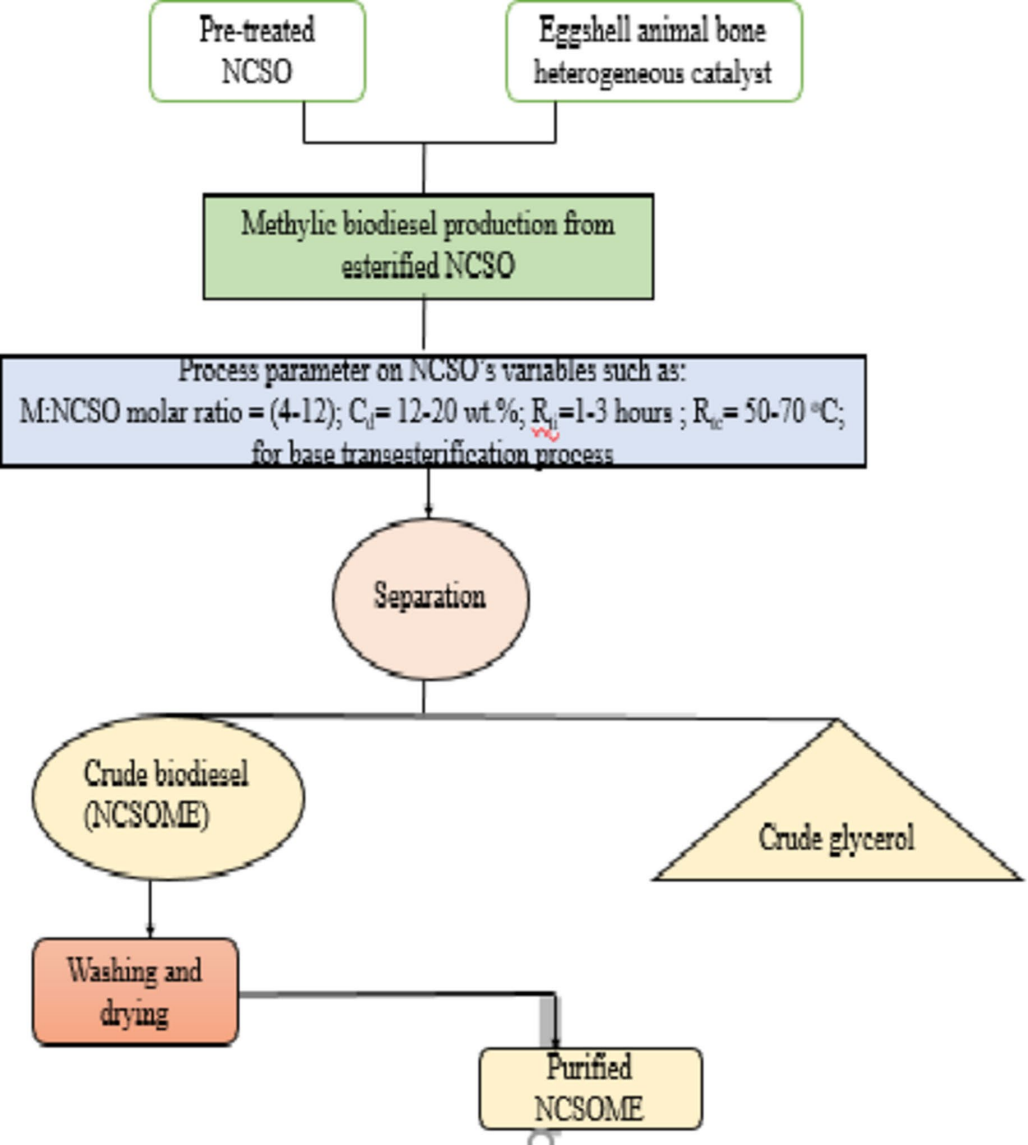
Production of TSOME from esterified NCSO.

**Fig. 2:**
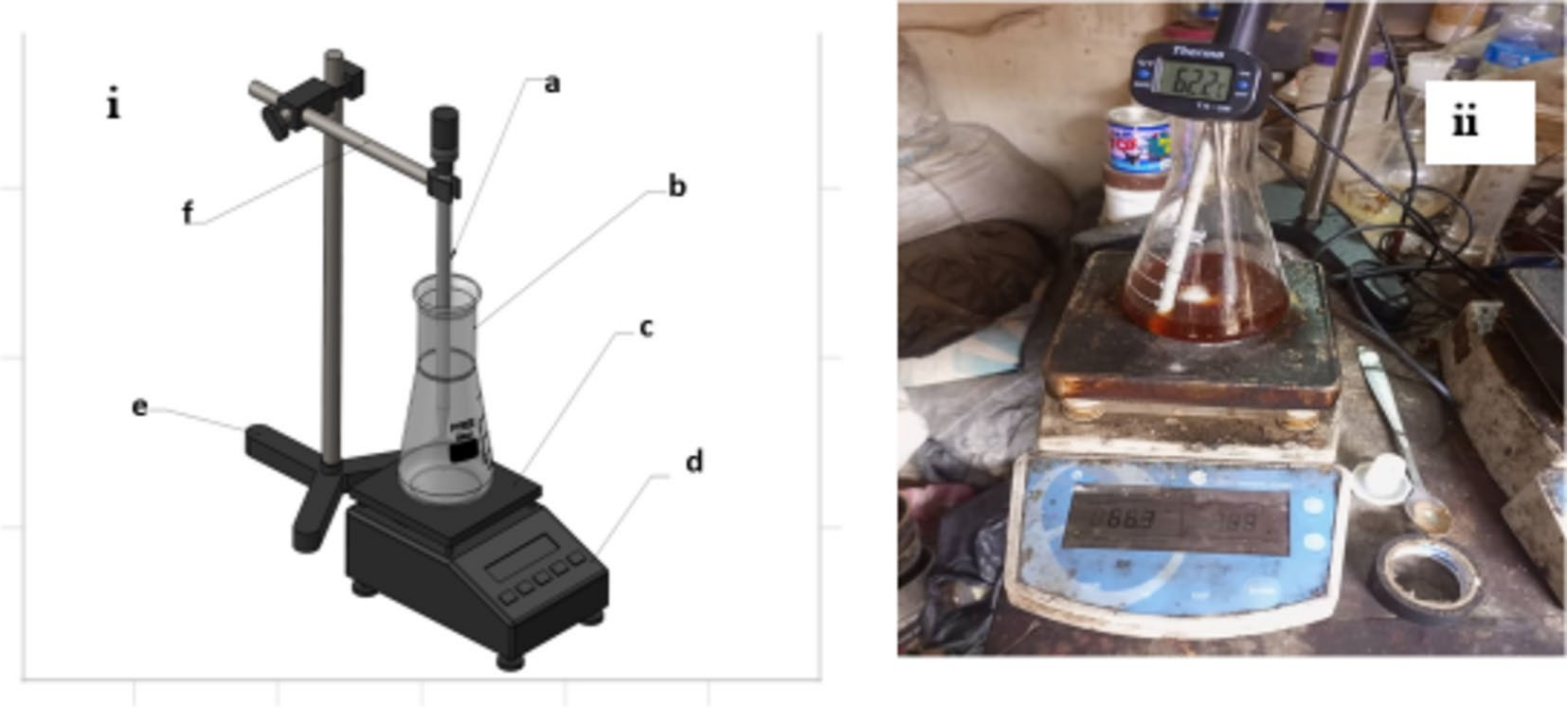
Biodiesel production process: (i) Schematic set-up and (ii) pictorial view of biodiesel processing equipment: (**a**) magnetic stirrer, (**b**) conical flask, (**c**) hot plate, (**d**) measuring device, (**e**) tripod stand, and (**f**) stirrer holder.

Figure 1 illustrates the biodiesel production process from pretreated NCSO. The pretreated NCSO undergoes basic transesterification as described previously. Figure 2(i-ii) shows the schematic and experimental set-up for composite biodiesel. An ECHC-methanol mixture is formed by mixing ECHC and methanol, which is then added to thermally esterified NCSO in a laboratory-scale reactor. After transesterification, NCSOME is allowed to settle. The KV was measured using a kinematic viscometer (see Table 3 and the procedure described elsewhere^67^. NCSOME yields and KV were determined for each experimental run.

### Preparation of a blend of ZnO NPs and NCSO biodiesel with varying ratios

**Fig. 3:**
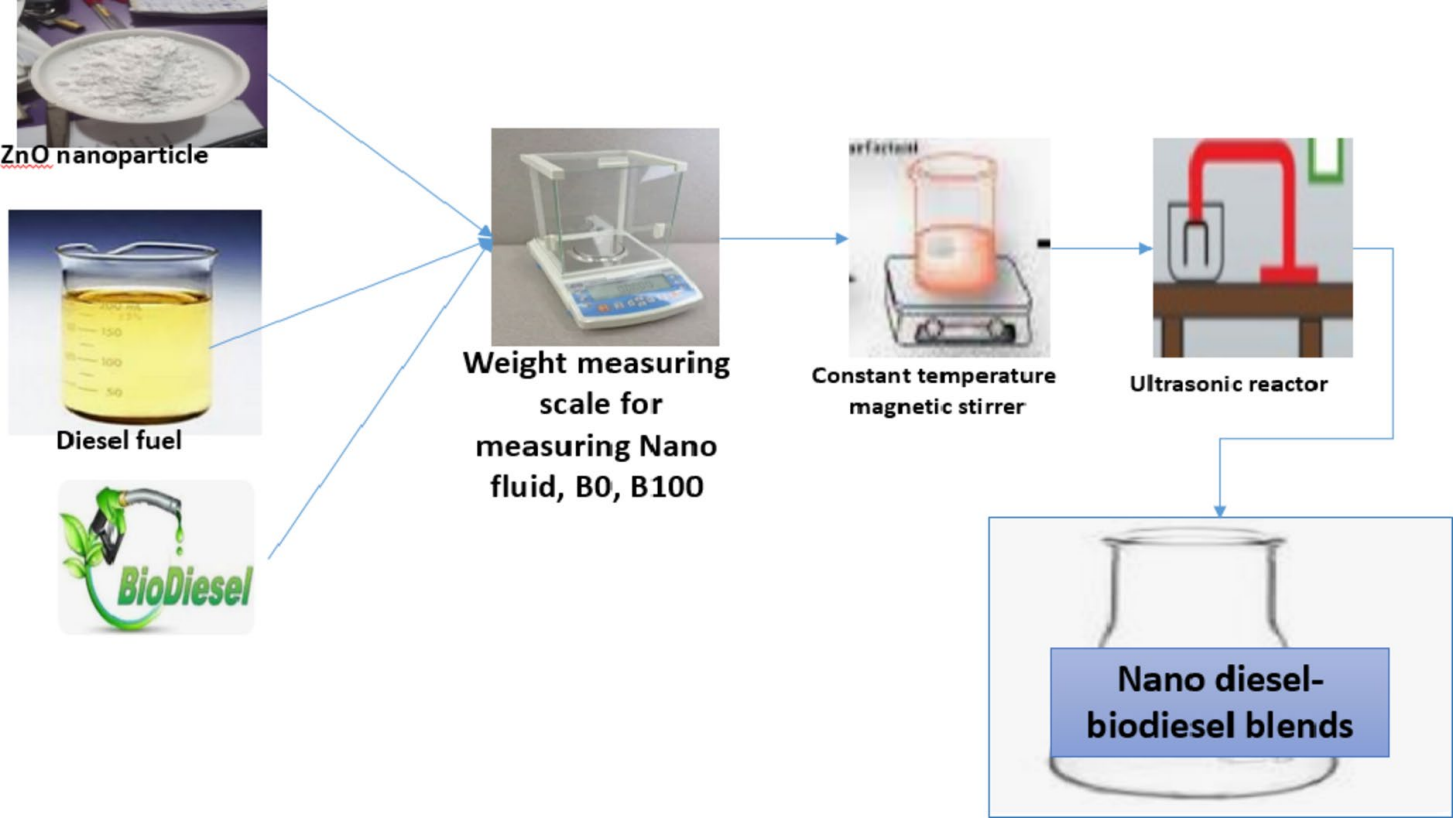
Schematic for the preparation of nano-fuels.

ZnO nanoparticles (30 nm, 99.9% purity) were obtained from a Nigerian laboratory. The nanoparticles were weighed using a sensitive scale (± 0.001 g) and mixed using a magnetic stirrer and ultrasonic process. Properties like density, viscosity, flash point, and calorific values were measured. Fuel blends (B0ZnO600, B10ZnO400, etc.) were prepared as shown in Fig. 3 and their results are summarized in Table 5.

**Table 5:** Characterization of NCSOME-nanoparticle with diesel blends.

Properties	Diesel	B0ZnO600	B10ZnO400	B10ZnO800	B20ZnO200	B20ZnO600	B20ZnO1000	B30ZnO400	B30ZnO800	B4OZnO600
Density (kg/m^3^)	830	824.10	823.10	832.40	836.60	832.88	833.10	829.70	830.30	833.10
Viscosity (mPa-s)	5.50	4.63	4.30	4.39	4.84	4.84	4.54	5.03	4.95	4.61
Flash point (K)	343	340	338	336	337	339	338	340	341	340
Calorific value (MJ/kg)	43.994	40.117	39.977	38.360	38.780	39.124	39.535	38.190	40.641	40.038

### Hybrid based models: NCSOME adopted input variable selection for ANN and ANFIS approach

Heterogeneous-based NCSOME was conducted in the lab-scale reactor as described in transesterification conditions^68–71^. Methylic variables (MVs) for the heterogeneous catalyzed biodiesel production derived from NCSO are methanol/oil molar ratio (4–10), catalyst amount (12–16 wt%), reaction time (1–3.0 h), and reaction temperature (50–70^°^C). The average yield was determined. The MVs were modelled using the ANN and ANFIS techniques to enhance NCSOME production.

#### ANN technique

**Fig. 4:**
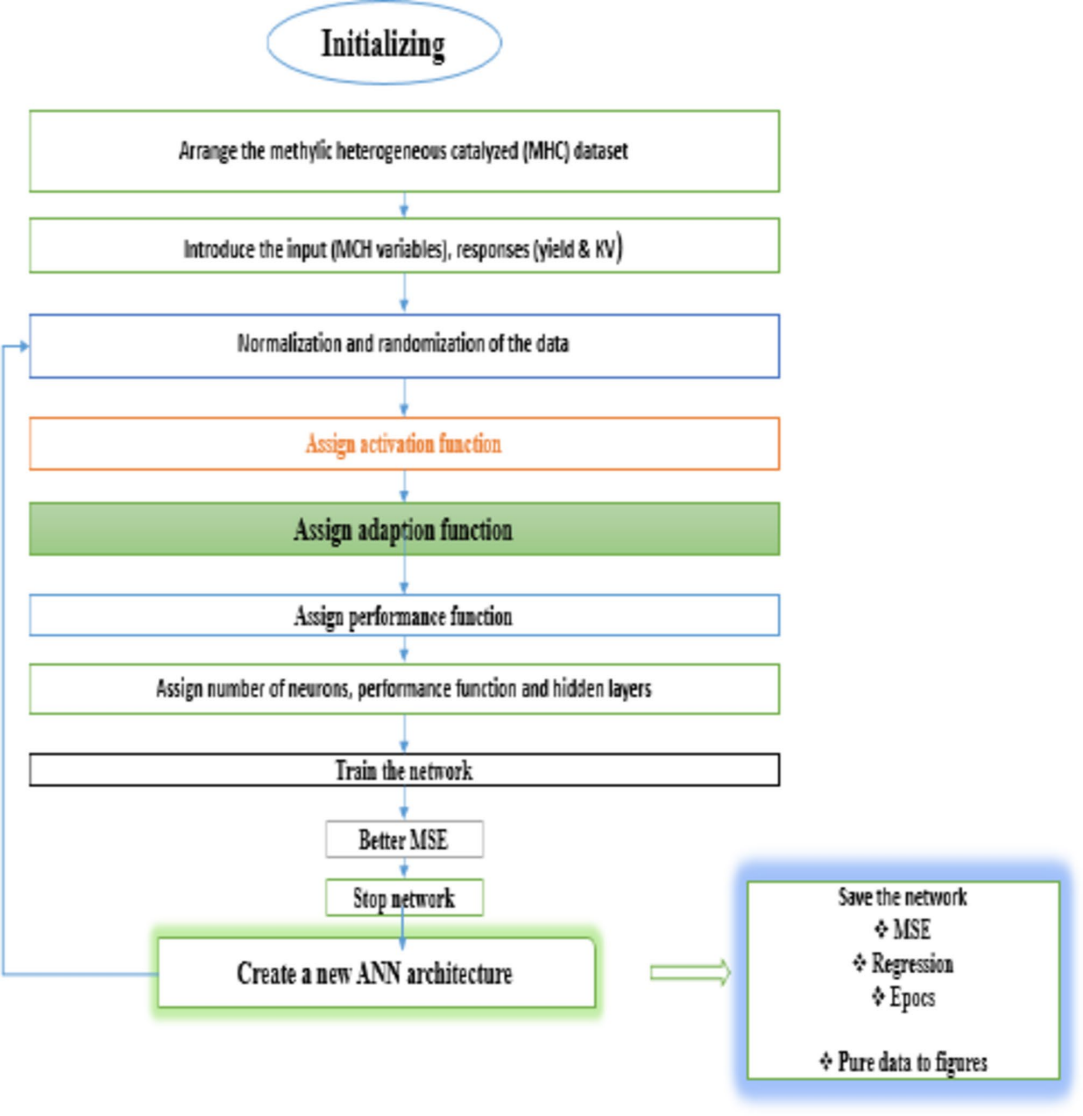
Flowchart of ANN network and data processing methods.

The ANN model was developed using MATLAB version 7.10 R2015a software (The Neural Network Toolbox, Inc., USA). The data set of CCD generated by RSM is used as input to the ANN model, where each data set represents all these methods together (See Table 6). In this study, a four-layer feedforward ANN model was developed, which consists of an input layer, hidden layer and output layer, respectively. The number of neurons in the input layer is 4 and the output layer is 2 for yield and kinematic viscosity. The values of network parameters for the ANN model are summarized in Table 7. The heterogeneous catalytic transesterification reaction program is different (methanol/esterification NCSO molar ratio, catalyst dosage, reaction time, reaction temperature). The data is divided into three groups: 70%, 15% and 15% of all data points are used for training, validation, and evaluation, respectively. The Levenberg-Marquardt algorithm is used for training the ANN model. Online learning is implemented using the Levenberg Marquardt algorithm. The ANN is retrained to minimize the mean square error (MSE) function of the ANN output and corresponding data. The gradient of MSE is used to adjust the network weights at each iteration. In this study, MSE is set as the stopping process at 10^− 4^, the minimum gradient is 10^− 10^, the maximum number of evidence is increased to 10 and the maximum number of epochs is set as 1000. The training process stops when one of these conditions is reached. Details of the ANN network for the ANN based modelling of NCSOME production are presented in Fig. 4.

**Table 6:** Experimental layout for NCSOME’s process parameters.

Runs	$\:{\partial\:}_{1}$	$\:{\partial\:}_{2}$ (wt%)	$\:{\partial\:}_{3}$ (h)	$\:{\partial\:}_{3}$ (°C)
R1	6	14	1.5	55
R2	10	14	1.5	55
R3	6	18	1.5	55
R4	10	18	1.5	55
R5	6	14	2.5	55
R6	10	14	2.5	55
R7	6	18	2.5	55
R8	10	18	2.5	55
R9	6	14	1.5	65
R10	10	14	1.5	65
R11	6	18	1.5	65
R12	10	18	1.5	65
R13	6	14	2.5	65
R14	10	14	2.5	65
R15	6	18	2.5	65
R16	10	18	2.5	65
R17	4	16	2.0	60
R18	12	16	2.0	60
R19	8	12	2.0	60
R20	8	20	2.0	60
R21	8	16	1.0	60
R22	8	16	3.0	60
R23	8	16	2.0	50
R24	8	16	2.0	70
R25	8	16	2.0	60
R26	8	16	2.0	60
R27	8	16	2.0	60
R28	8	16	2.0	60
R29	8	16	2.0	60
R30	8	16	2.0	60

$\:{\partial\:}_{1}$= Methanol/esterified NCSO molar ratio, $\:{\partial\:}_{2}$ = Catalyst amount (wt%), $\:{\partial\:}_{3}$ = Reaction time (h), $\:{\partial\:}_{4}$ = Reaction temperature °C.

**Table 7:** Network parameters for ANN model.

Parameters	ANN
Number of input layer units	4
Number of hidden layer	1
Number of hidden layer units	4–16
Number of output layer units	1
Transfer function in hidden layer	tansig
Transfer function in hidden layer	Purelin
Training function	Levenberg-Marquardt
Learning rate	0.01
Performance goal	0
Maximum number of epoch	1000

#### ANFIS based modelling of NCSOME

**Fig. 5:**
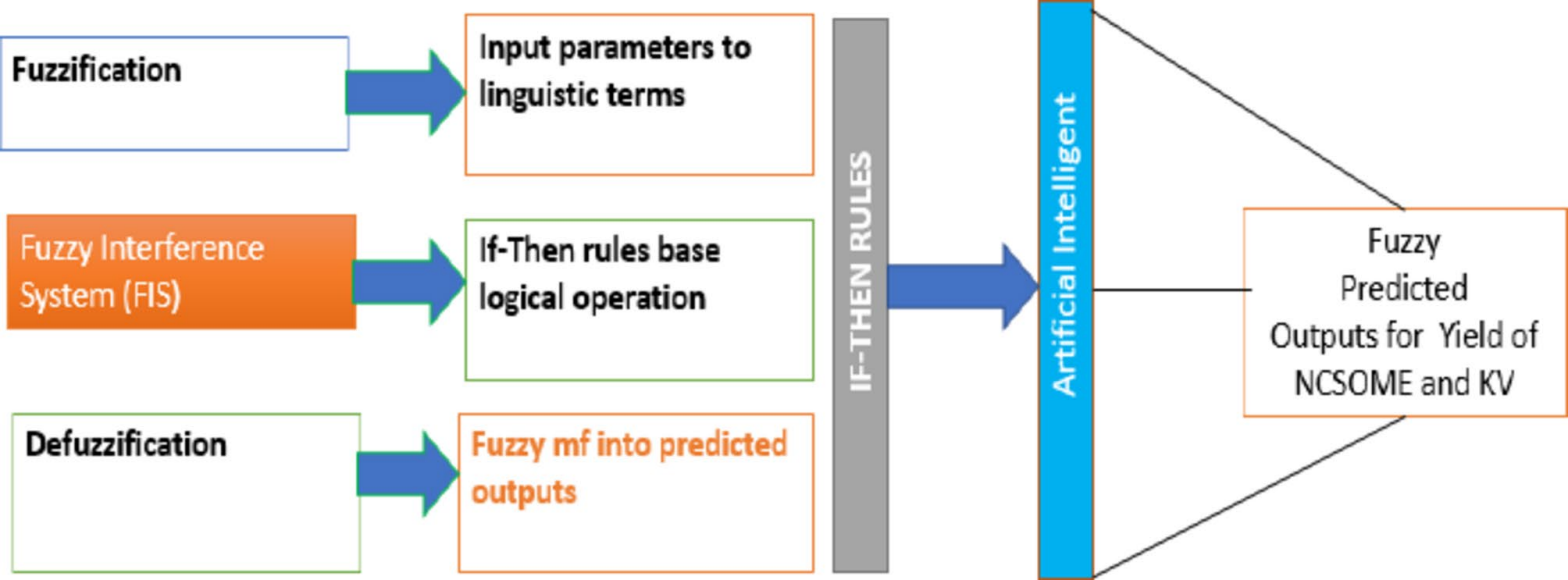
Flowchart for ANFIS.

The ANFIS-based models were created using the anfis-edit tool in MATLAB Version 7.10 R2015a. Different input parameters such as methanol/NCSO molar ratio, catalyst amount, reaction time, and temperature were used for various predictions. The modelling process for the heterogeneous catalysed transesterification protocol to produce NCSOME with the ANFIS method involves several steps, including training and testing. The methodology for ANFIS modelling is shown in Fig. 5. Further details on the theory, state-of-the-art, and mathematical computations related to ANFIS can be found in other sources^17,52,71^.

## Methodology for assessing environmental attributes or emission features

**Fig. 6:**
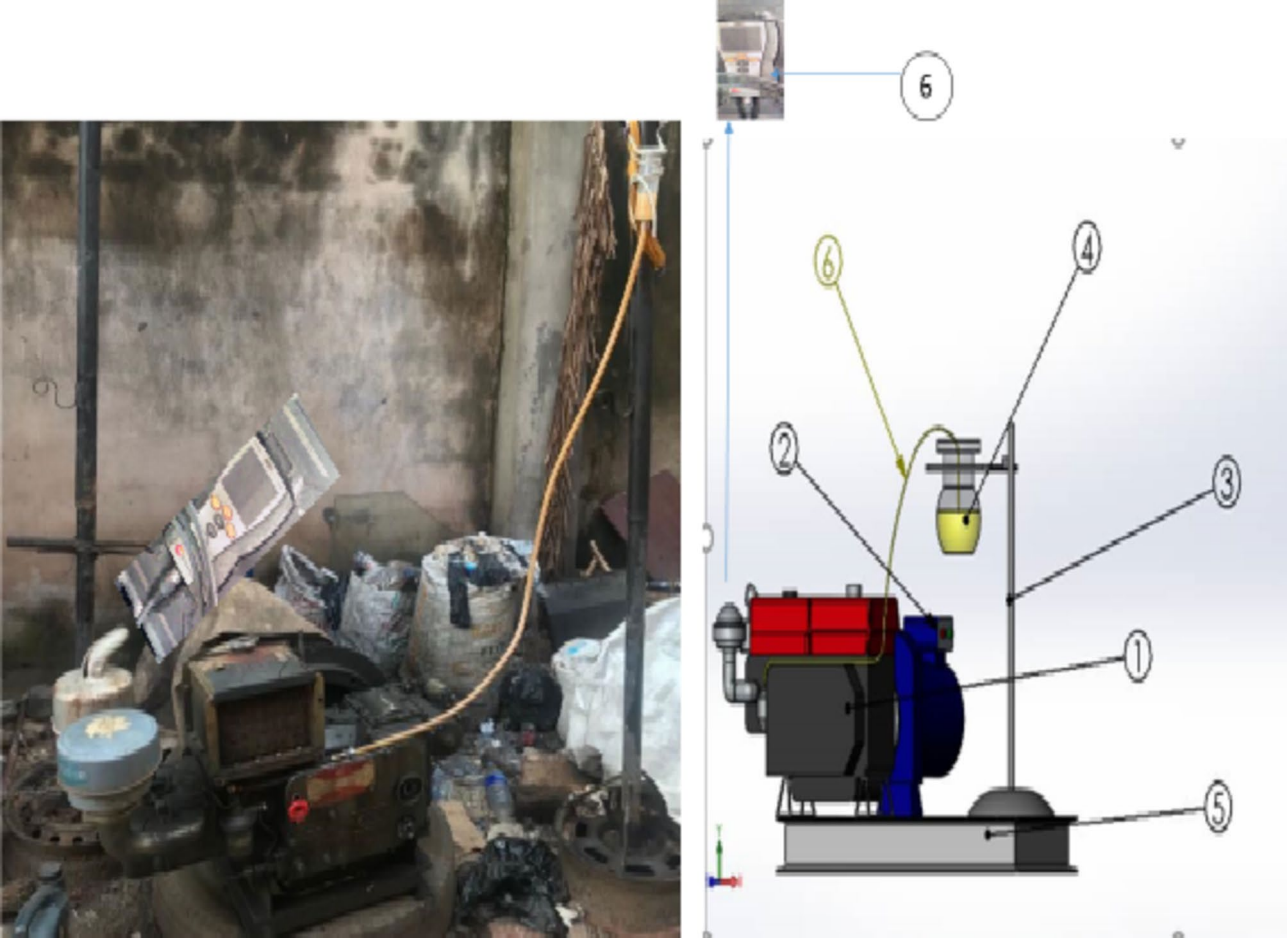
Diesel engine set-up for determining emission tail profile: (**a**) pictorial view, (**b**) schematic representation: (1) diesel engine unit, (2) generator unit, (3) container support, (4) fuel adopted, (5) base standard, (6) Testo gas analyzer.

Environmental characteristics, such as exhaust emission profile (EEP), were evaluated on a direct injection (DI) single-cylinder, water-cooled, four-stroke, naturally aspirated test diesel engine model (Zhejiang Sifang). The DI diesel engine was linked to a hydraulic dynamometer to generate different loads. Figure 6 (a-b) shows the visual representation and schematic setup of the diesel engine used for emission testing. The Testo 350 XL gas analyzer was employed to measure EEP. The complete configuration and specifications of the gas analyzer are described elsewhere^73,74^.

**Fig. 7:**
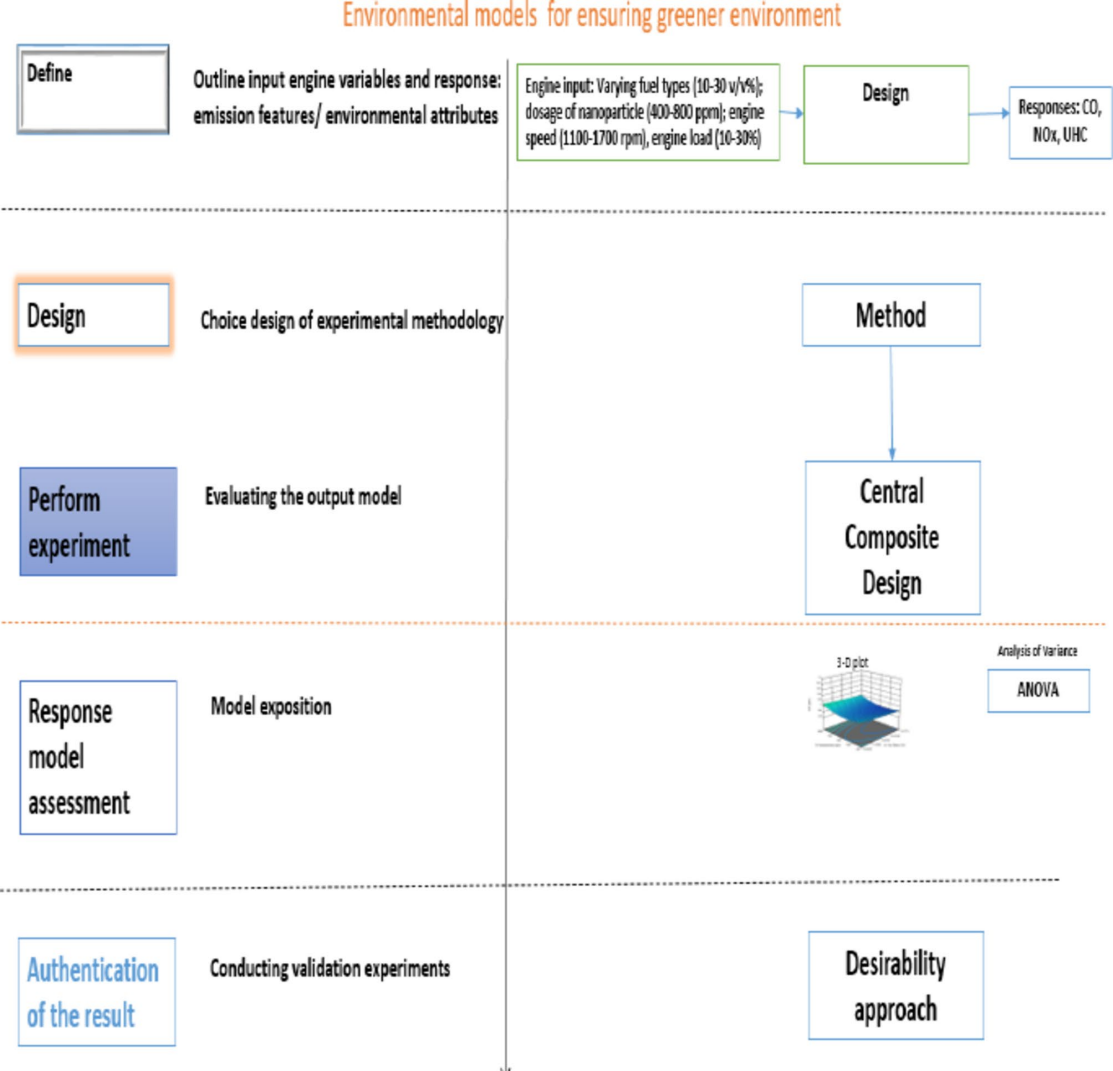
Graphic flow for the key emission profile based modelling.

To ensure consistent results, the engine was operated for a steady state period of approximately thirty-five minutes using pure diesel before each new experiment with the tested fuel. Experimental values for carbon monoxide (CO), nitrogen oxide (NO_x_), and unburnt hydrocarbon (UHC) were measured for different fuel types (10–30 wt%), nanoparticle dosages (400–800 ppm), engine speeds (1100–1700 rpm), and engine loads (10–30%) using a design of experiment (DOE) based on the response surface methodology (RSM) layout shown in Fig. 7. The input variables, including the nanoparticle dosage range of 400–800 ppm, were selected based on relevant literature^75–77^, while those of engine speed were based on studies discussed elsewhere^78,79^.

Thirty experiments were conducted following the DOE of RSM. Emission parameters such as CO, NO_x_, and UHC were recorded as environmental attributes for analysis. Two readings were taken for each experimental observation to ensure measurement accuracy, and the average values were recorded. The recorded emission data were used for conducting an analysis of variance (ANOVA). Table A1 in the Appendix/Supplementary A highlights the datasets for analysing the emission tail profile.

### Uncertainty evaluation of low carbon and its compliance with the environment

The uncertainty analysis aims to examine the compliance of low carbon fuel operated in an IC engine with environmental standards. Various factors can introduce errors naturally, such as instrument conditions, laboratory settings, instrument calibration, environmental factors, and measurement readings. The uncertainty analysis of the measuring equipment followed the procedure outlined in the literature^80^. The overall uncertainty analysis was conducted using Eq. (1) and the results are summarized in Table 8.

**Table 8:** Uncertainty valuation of test engine.

Measuring variables	Uncertainty
CO	1.61365
NO	0.01832
UHC	0.04641

1 $$\begin{aligned} & \:{\text{Agregate}}\:{\text{uncertainity}} \\ & = ({\text{Uncertainity}}\:{\text{of}}\:{\text{CO}})^{2} + ({\text{Uncertainity}}\:{\text{of}}\:\:{\text{NOx}})^{2} \\ & + ({\text{Uncertainity}}\:{\text{of}}\:{\text{UHC}})^{2} \\ & \:{\text{Agregate}}\:{\text{uncertainity}} = {\text{square}}\:{\text{root}}\:[(1.61365)^{2} + (0.01832\:)^{2} + \left( {0.04641)^{2} } \right] \\ & = 1.61442\% \\ \end{aligned}$$

### Model appraisal methodology

Statistical variables such as regression coefficient (R^2^), mean average error (MAE), standard error and absolute average deviation (AAD) were adopted to determine the superiority and predictive ability of the model techniques. Equations (2)–(4) were employed to determine the statistical indices of the RSM and ANN models. The superiority and efficacy of the model techniques were determined based on the results.

2 $$\:{R}^{2}=1-\frac{\sum\:_{i=1}^{n}({Z}_{i,p}-{Z}_{1,e}{)}^{2}}{\sum\:_{i=1}^{n}({Z}_{i,p}-{Z}_{e,\:ave}{)}^{2}}$$

3 $$\:{\text{MAE}} = \sum\limits_{{{\text{i}} = 1}}^{{\text{n}}} {\frac{{\left\lfloor {({\text{Z}}_{{{\text{i}},{\text{e}}}} - {\text{Z}}_{{{\text{i}},{\text{p}}}} )} \right\rfloor }}{{\text{n}}}}$$

4 $$\:{\text{AD}} = \frac{{100}}{{\text{n}}}\sum\limits_{{{\text{i}} = 1}}^{{\text{n}}} {\frac{{\left\lfloor {({\text{Z}}_{{{\text{i}},{\text{e}}}} - {\text{Z}}_{{{\text{i}},{\text{p}}}} )} \right\rfloor }}{{\left( {{\text{Z}}_{{{\text{i}},{\text{e}}}} } \right)}}}$$

## Results and discussion

### Basic properties of suitable NCSO and its fatty acid compositions

Tables B1 and B2 compare the properties of castor seed oil (CSO), neem seed oil (NSO), their blends (N10, N20, N40, N50) and those in the literature RSO50F050^81^, CI20CP80^82^, and NSO 20CSO80^83^ (See Appendix/Supplementary B). The oil contents of the castor seed and neem seed were found to be 52.1% and 30–39%, respectively, which are much higher than those reported from other vegetable seeds^84,85^. Hence, CSO and NSO are prospective feedstock candidates for BD production. As observed in Table A.I, the thermophysical properties (TPs) of CSO have been modified with the NSO fraction in the prepared blends. The modification is attributed to the lower AV, SV, density, and kinematic viscosity of NSO in contrast to the CSO. The major TPs of the blends align with those of RSO50F050^81^, CI20CP80^82^, and NSO 20CSO80^83^. Prior methylic composite biodiesel production from the selected blend CSO + NSO, N20 was selected for pre-treatment after a parametric study. The esterification protocol and optimal conditions for the selection of the said blend to conduct transesterification are discussed elsewhere^86^.

**Fig. 8:**
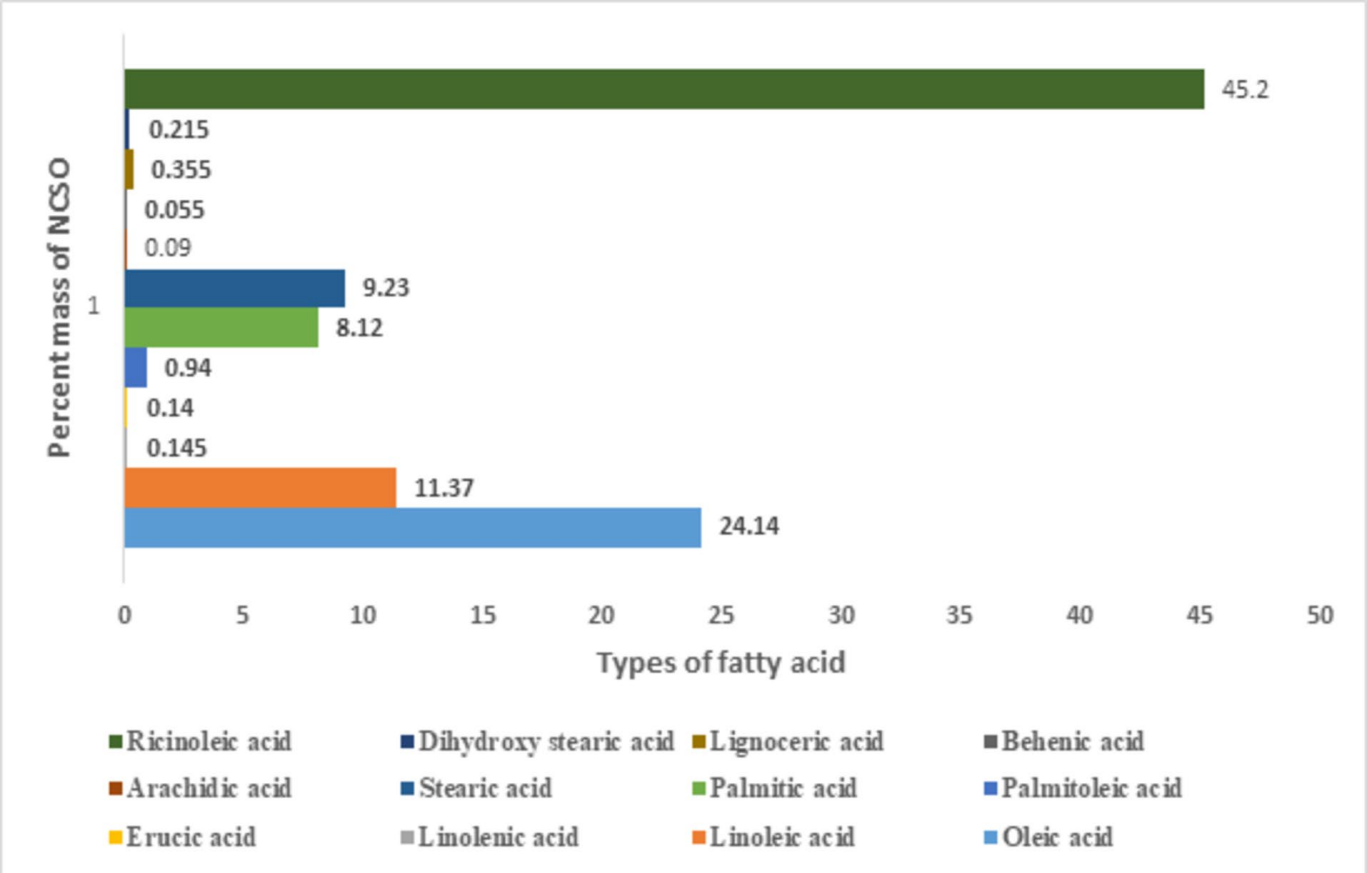
Fatty acid compositions of neem-castor seed oil.

The fatty acid composition of N20 is depicted in Fig. 8. As observed, apart from the highest component of ricinoleic acid (45.2%), oleic acid (24.14%), stearic acid (9.23%), then palmitic acid (8.12%). While others in a minute percentagerange from 0.09 to 0.355 with arachidic acid being the lowest. The high percentage of unsaturated level of NSCO operated on a diesel engine exhibits higher advancements in fuel injection timing, ignition delay, heat release rate (HRR), maximum gas pressure, and exhaust gas temperature^87^.

### Characterisation of the produced catalysts

**Fig. 9:**
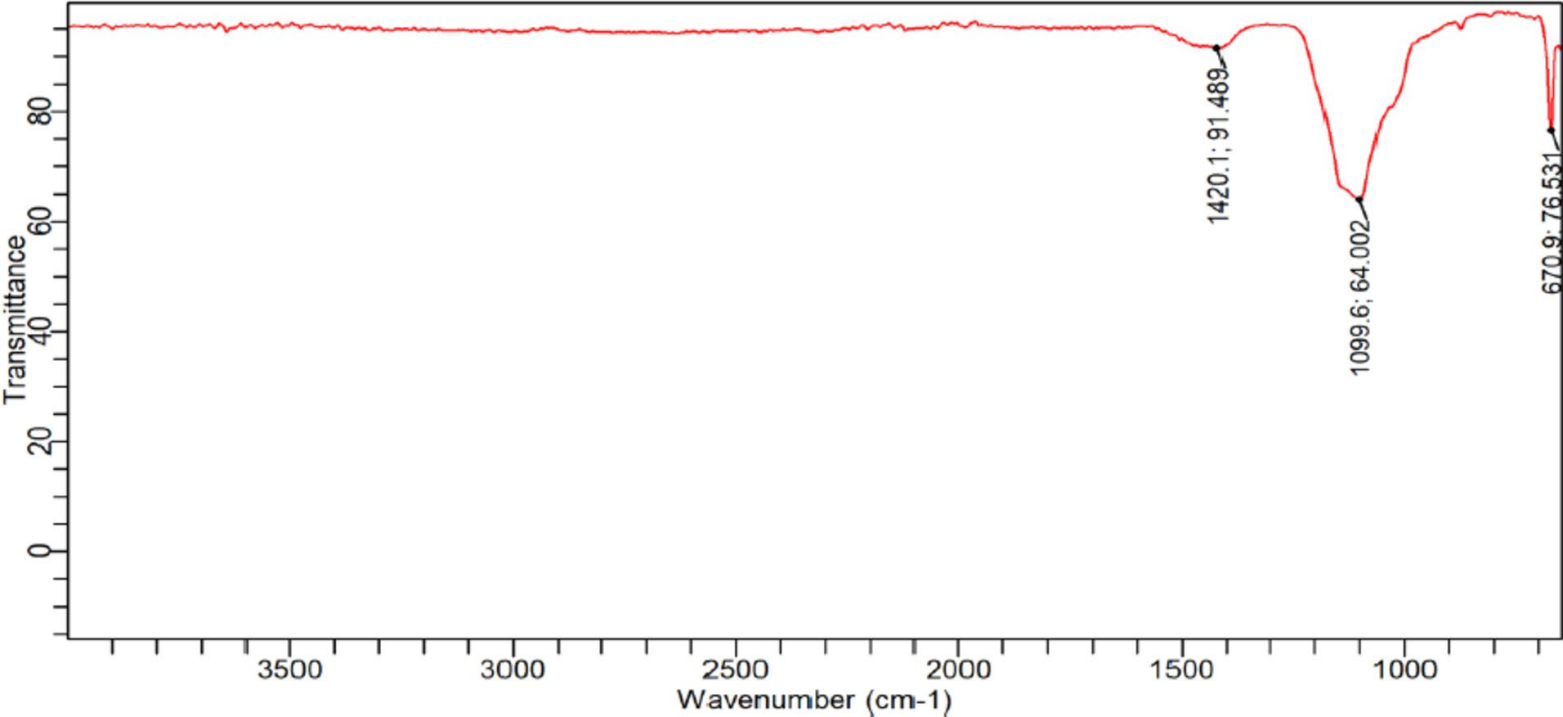
FT-IR analysis of heterogeneous catalyst synthesized from eggshells and animal bones.

Figure 9 depicts the FTIR spectrum of eggshells and animal bones-based HEC conducted to detect the presence of functional groups on the surface of the synthesized catalyst. The FT-IR spectra were observed in the 100 cm^− 1^ to 1500 cm^− 1^ range. The highest peak that developed at 1420.11572 cm ^− 1^ is attributed to the carbonate ion, whereas the bands positioned at 1099.56466 cm ^− 1^ and 670.92081 cm^− 1^ suggest sulfate ion and aryl thioethers (ϕ – S) (C – S stretch), respectively. As observed, the maximum peak of the spectrum elucidates the efficient formation of the support material for the heterogeneous catalyst formation.

### Modelling of NCSOME through ANN and ANFIS techniques

#### Modelling using ANN technique

**Fig. 10:**
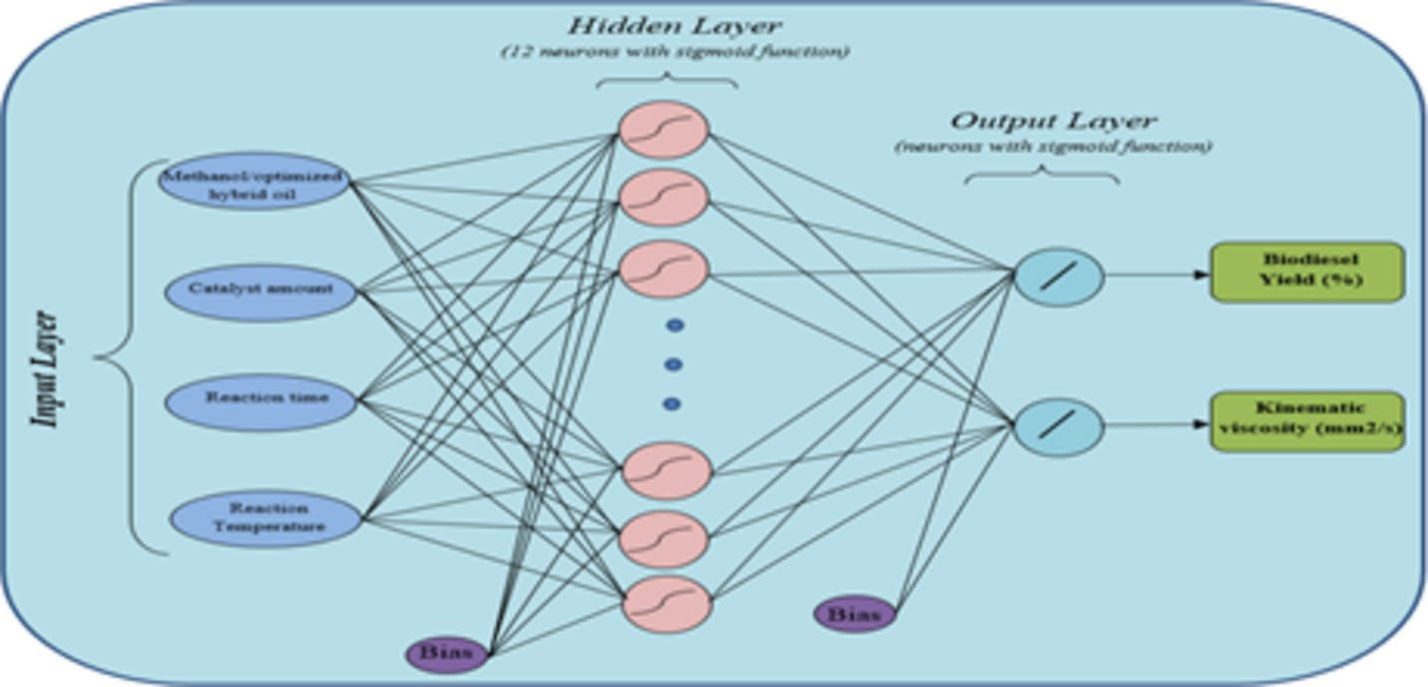
The topology of ANN.

The ideal prediction is determined by the architecture of the ANN^88^. Figure 10 potrays the graphic of a multilayer artificial neural network. As can be seen, the input layer consists of four parameters in the process (methanol/optimized hybrid ratio, catalyst amount, reaction time, reaction temperature), twelve hidden layers, and the output contains two layers, which are biodiesel yield (BY) and KV of transesterified NCSO. The optimal condition is obtained by trial and error.

The selection of relevant neurons depends on the specified criteria. The output system is divided into two groups: BY and KV. The preference for layers with a small number of nodes is due to the reduction of the network size and the increase in information capacity^89^.

**Fig. 11:**
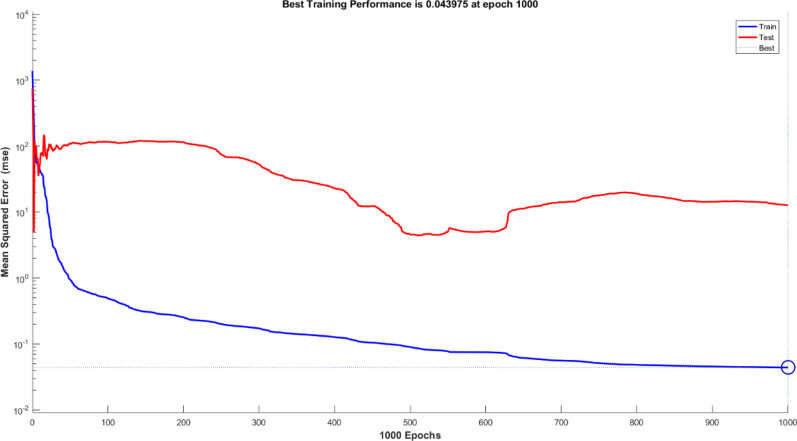
Plot of SSE against training subset and test subset.

To maintain the optimal network structure, many clusters with varying numbers of nodes were created. Figure 11 shows the MSE curves for the training and testing subsets. As observed, the hidden layer with eight nodes and the lowest MSE value yielded the best estimation.

**Fig. 12:**
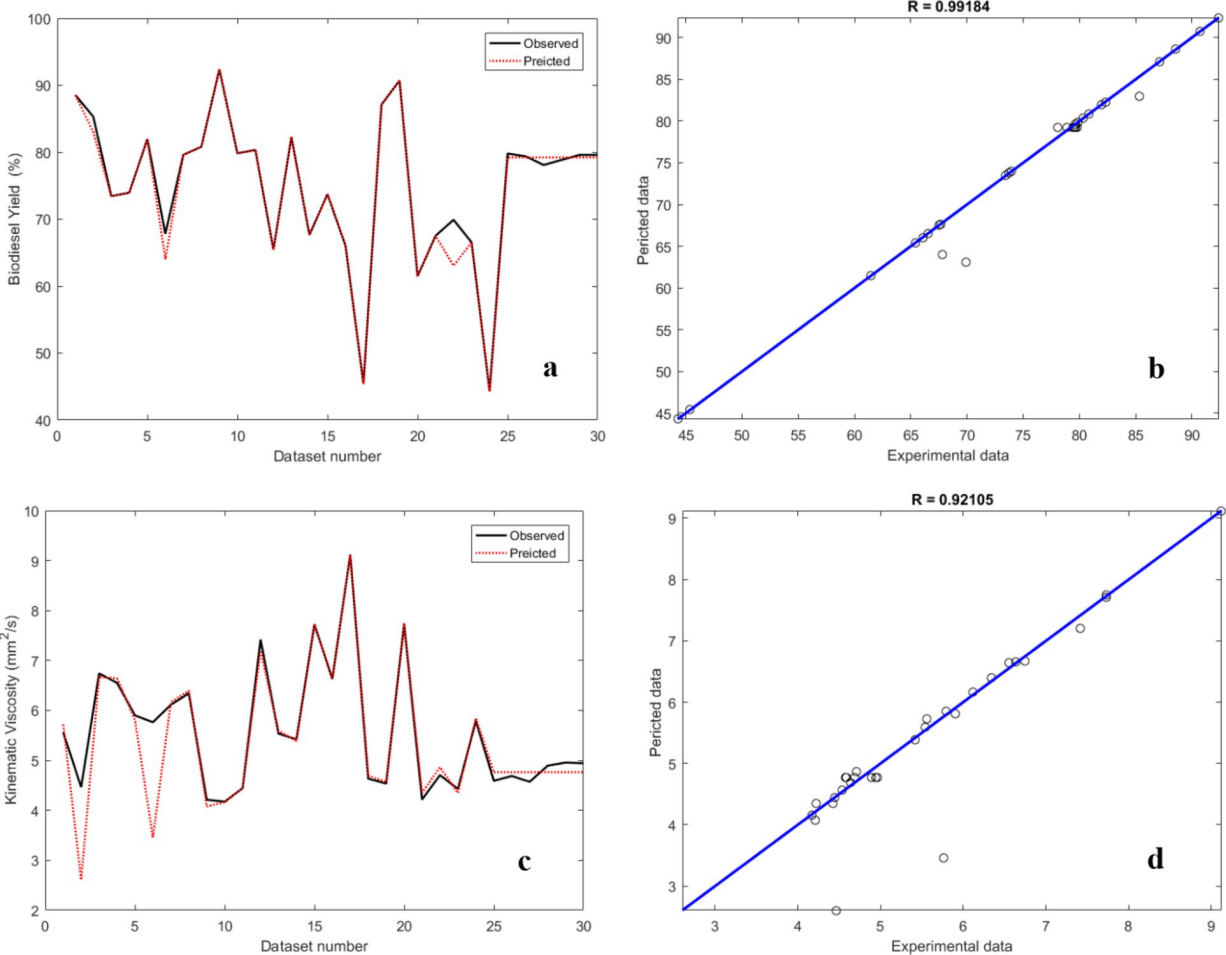
The performance of ANN model for biodiesel yield and KV prediction: (**a**): network for measured and predicted biodiesel yield, (**b**) MLP model for biodiesel yield, (**c**), network for measured and predicted KV, and (**d**) MLP model for KV.

Figure 12 (a-d) shows the biodiesel yield network, the biodiesel yield predicted by a multilayer perceptron (MLP) model, the biodiesel yield network and the MLP model for KV. The correlation coefficient (R) of the proposed MLP model for biodiesel (0.99184) and the correlation coefficient for KV (0.92105) show that the output and target predictions of the artificial neural networks for each sample are in good agreement.

#### Modelling using ANFIS technique

**Fig. 13:**
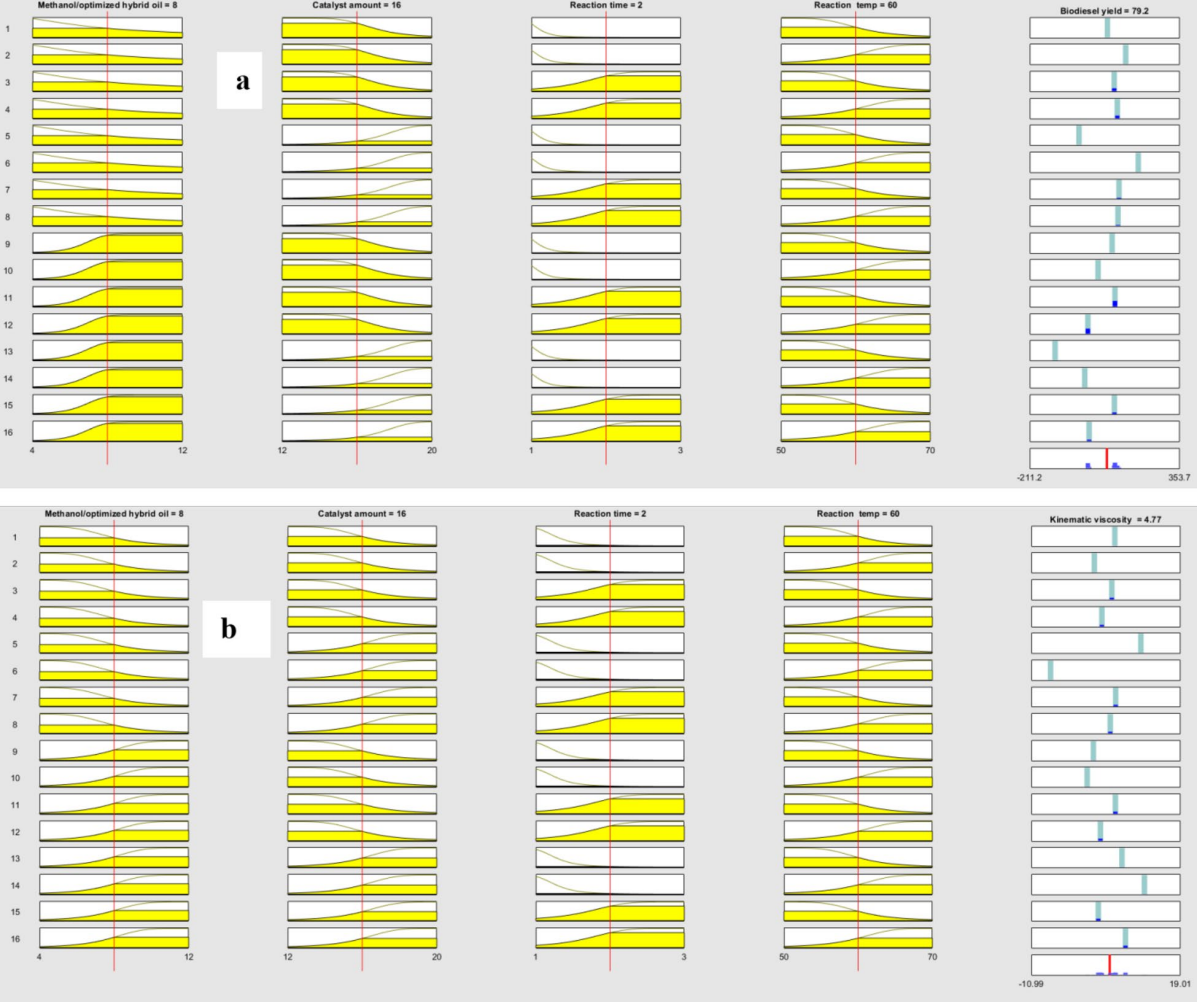
Fuzzy rule viewer: (**a**) BD and (**b**) KV.

Figure 13 (a-b) depicts rule viewers that show value of the various transesterification parameters (TPs) in the ANFIS models and computed yield of biodiesel (YoB) and KV. The YoB and KV can be predicted by varying the TPs, including methanol/hybrid esterified oil molar ratio, catalyst amount, reaction time, and reaction temperature in the developed ANFIS models.

**Fig. 14:**
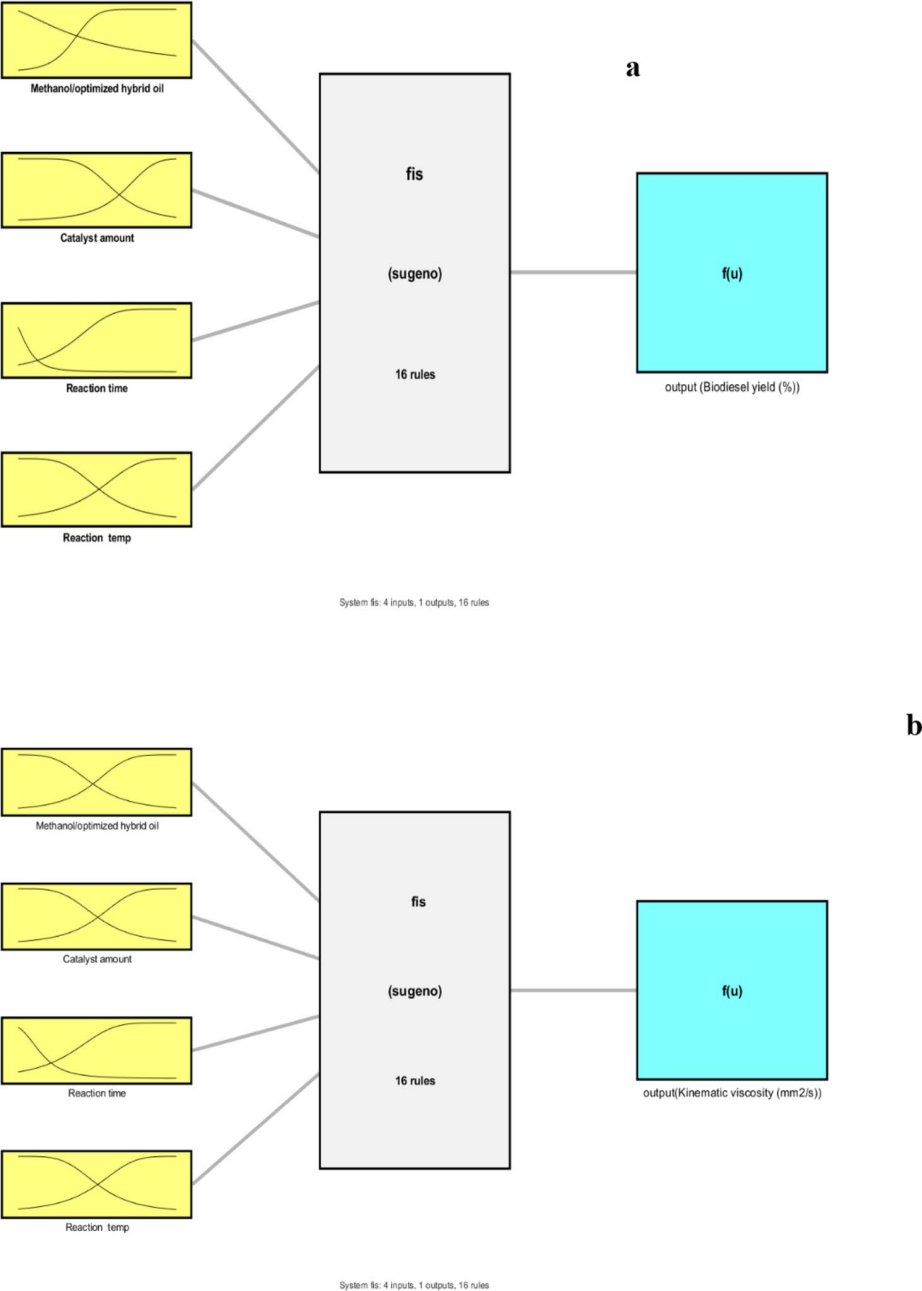
Developed fuzzy interference system: (**a**) yield and (**b**) kinematic viscosity.

Figure 14 (a-b) depicts the architecture of the developed ANFIS model for YoB and KV, respectively As oberved, fuzzy systems with 55 nodes, 80 linear parameters, 24 non-linear parameters, 16 fuzzy rules, and 16 inference rules for each input were sufficient to represent process performance.

#### Comparison of ANN and ANFIS models for BD’s yield and its KV

**Fig. 15:**
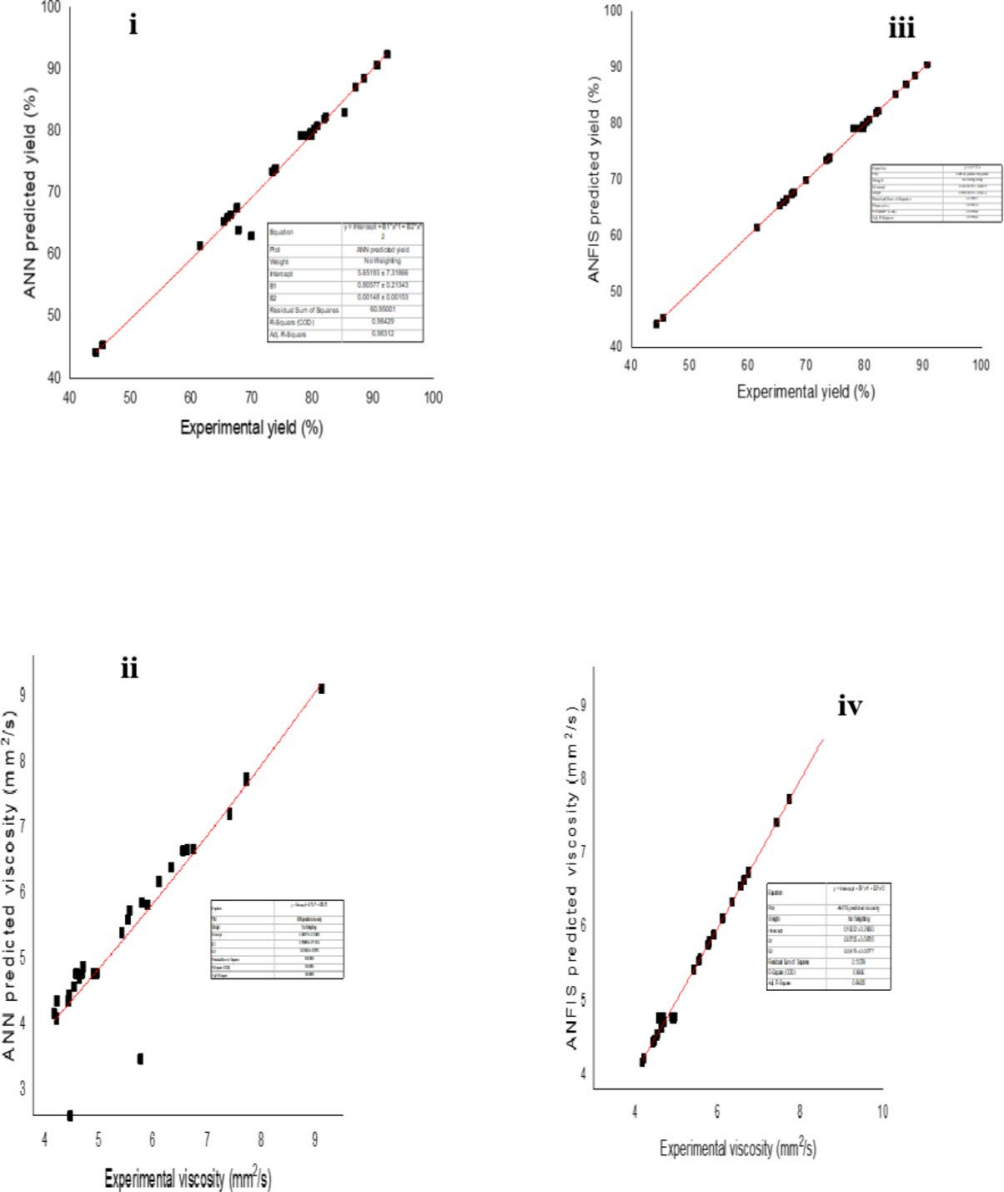
Contrast of the various biodiesel yield and KV: (i) ANN predicted and experimental yield, (ii) ANN predicted and experimental KV, (iii) ANFIS predicted and experimental yield, and (iv) ANFIS predicted and experimental KV.

Figure 15 (i, ii) show the comparison between the predicted yield and actual data for yield and KV using ANN, while Fig. 15 (iii, iv) display the comparison between the predicted yield and actual data for yield and KV using ANFIS for NCSOME. As demonstrated by the hybrid models (HMs), the dispersion of data points in the ANFIS-based predicted yield and KV is greater than in the ANN model. Regarding the HMs, the ANFIS model demonstrated a better fit to the actual/experimental data compared to the ANN model. This was also confirmed by the appraisal metrics highlighted in Table 9. The ANFIS model showed superior performance in terms of R, R^2^, RMSE, SEP, MAE, and AAD compared to the ANN model in predicting yield and KV for NCSOME.

**Table 9:** Appraisal metrics for soft computing based modelling of yield and KV of NCSOME.

Variables	ANN model		ANFIS model	
	NCSOME yield (%)	KV (mm^2^/s)	NCSOME yield (%)	KV (mm^2^/s)
R	0.9918	0.9212	0.9997	0.9983
R^2^	0.9837	0.8486	0.9994	0.9966
RMSE	1.5164	0.4720	0.2619	0.0728
MAE	0.5347	0.2228	0.0997	0.0314

#### Fuel properties of NCSOME

The synthesised NCSOME must satisfy the requirements outlined in ASTM D 6751 and EN 14,214 guidelines in order to be evaluated for commercial viability. The thermophysical properties were analysed after a comprehensive parametric study of methylic transesterification conditions (methanol/oil = 9.9; catalyst dosage = 14.1; reaction time = 59.1 min; yield = 92.5% and KV of 4.131) as indicated elsewhere^90^.

Table 10 highlights the properties of NCSOME and those of other biodiesels. As observed, the density of NCSOME (808.2 kg/m^3^) complied with the ranges of the EN 14,214 standard (860–900 kg/m^3^) and compared favourably with those of Mallah and Sahito^91^ (896 kg/m^3^) and CAOME (912.4 kg/m^3^). However, it was slightly higher than that of diesel (B0) (860 kg/m^3^). NCSOME having higher density compared to B0 will not significantly influence brake fuel consumption when injected^92^.

**Table 10:** Fuel properties of the NCSOME at optimal condition.

Property	NCSOME*	NCSOME (Mallah and Sahito^91^)	CAOME Thakkar et al.^93^	ASTM D6751	EN 14,214	B0
Density, 40 °C (kg/m^3^)	808.2	896	912.4		860–900	860
Kinematic viscosity, 40 °C (mm^2^/s)	4.128	5.83	11.31	1.9–6.0	3.5–5.0	4.012
Flash point (°C)	121	160	140.5	130 min	120 min	77
Acid value (mgKOH/g)	0.38	0.3		0.50 max	0.50 max	0.13
Cloud point (°C)	6.9	5	− 25	–	–	− 8
Pour point (°C)	3.4	− 4	− 45	< 0	< 0	− 14

*Present study.

CAOME = castor oil methyl ester.

The flash point (FPT) of NCSOME (121 °C) agreed with those of Mallah and Sahito^91^ (160 ^o^C) and Thakkar et al.^93^ (140.5 ^o^C) but higher than that of B0 (77 °C), however, it meets safety norms for both international standards. Biodiesel with a high FPT is less disposed to fire threats compare to diesel fuel^86^.

NCSOME displayed CP and PP values of 6.9 °C and 3.4 °C, which are higher than those of B0 (− 8 °C and − 14 °C, respectively). These high values of PP and CP are attributed to the abundance of saturated fatty esters in biodiesel and might limit its wider application in cold regions^80^.

Although NCSOME’s acid value (AV) of 0.38 mg KOH/g was higher than Mallah and Sahito^91^ (0.3 mg KOH/g, it nevertheless met ASTM D6751 and EN 14214’s (0.50 mg KOH/g) requirements. Due to the fuel’s low acid value, NCSOME undergo through polymerization^52^.

## Regression models for NCSOME-diesel IC engines

Emission features of IC engines operated on NCSOME/diesel blends were evaluated for a sustainable and cleaner environment using an experimental design matrix. These models were analysed, evaluated and optimized for the desired criteria of minimising environmental features namely CO, NO_x_, and UHC. To examine the environmental aspects, three critical parameters—CO, NOx, and UHC—were chosen. Prior to using these mathematical formulas to forecast emission properties, ANOVA was used to establish their correlation function with the inputs, or control factors. Table 11 highlights the significant impact of factors on responses based on p-values. As can be observed, the quadratic of engine speed p-values are the most effective compared to linear engine speed and nanoparticle dosage.

**Table 11:** P-values of model terms.

Factors	A	B	C	D	AB	AC	AD	BC	BD	CD	A^2^	B^2^	C^2^	D^2^
CO	0.3236	0.1876	**0.0030**	**< 0.0001**	0.2305	0.8284	0.4313	**0.0657**	**0.0499**	0.0657	0.6261	0.3915	**< 0.0001**	**0.0090**
NOx	0.7906	**0.0021**	**0.0033**	0.5193	0.1390	0.7681	0.5132	0.8205	0.7971	0.8205	0.9578	0.2439	**0.0004**	0.8766
UHC	0.6655	0.0981	0.2056	0.2876	1.00	0.1969	0.4305	0.1969	0.4305	0.2971	**0.0021**	**0.0021**	**0.0181**	0.3907

Significant- ($$\:0.000<p\le\:0.05)$$

(A: Fuel types/blends, B: Nanoparticle’s dosage, C: Engine speed, D: Load, AB: Fuel types x nanoparticle’s dosage, AC: Fuel types x engine speed, AD: fuel type x load, BC: Nanoparticle’s dosage x engine speed, BD: Nanoparticle’s dosage x load, CD: Engine speed x Load, A^2^: Quadratic of fuel types, B^2^: Quadratic of nanoparticle’s dosage, C^2^: Quadratic of engine speed, D^2^: Quadratic of load).

The emissions of various IC engines can be evaluated using regression equation itemized in Table 12. Equations (5a) -(5c) can correctly hypothesize CO, NOx, and UHC.

**Table 12:** Regression equations of responses.

Regression equations for emission profile	
CO	$$\begin{aligned} & 53.0 - 0.7083A - 0.9583B + 2.46C + 6.29D + 1.06AB - 0.1875AC - 0.6875AD \\ & \quad + 1.69BC - 1.81BD + 1.69CD + 0.3229A^{2} + 0.5729B^{2} + 5.20C^{2} + 1.95D^{2} \\ \end{aligned}$$ (5a)
NOx	$$\begin{aligned} & 0.5450 + 0.0036A - 0.0491B + 0.0463C - 0.0088D - 0.0254AB + 0.0049AC \\ & \quad + 0.0109AD - 0.0037BC + 0.0043BD + 0.0038CD - 0.0007A^{2} \\ & \quad + 0.0150B^{2} + 0.0566C^{2} + 0.0020D^{2} \\ \end{aligned}$$ (5b)
UHC	$$\begin{aligned} & 2.77 - 0.0167A + 0.0667B + 0.050C + 0.0417D + 0.00AB + 0.0625AC \\ & \quad + 0.0375AD - 0.0625BC + 0.0375BD + 0.050CD + 0.1312A^{2} + 0.1312B^{2} \\ & \quad + 0.0938C^{2} + 0.0312D^{2} \\ \end{aligned}$$ (5c)

### Emission models

The interaction of control factors, which are engine input variables, with response variables such as BTE, BSFC, CO, NOx, and UHC, was studied. Emission variables like CO, NOx, and UHC were analyzed and represented using 3-D response surface diagrams.

#### CO model

Table 12 highlights the terms and interactions of engine inputs and the CO model. As observed, all the terms are significant for the CO model except fuel types, nanoparticle dosage, the interaction of fuel types and nanoparticle dosage, fuel types and engine speed, nanoparticle dosage and engine speed, quadratic of fuel types, and nanoparticle dosage. The correlation of the CO model and engine input is represented with Eq. (5a).

**Fig. 16:**
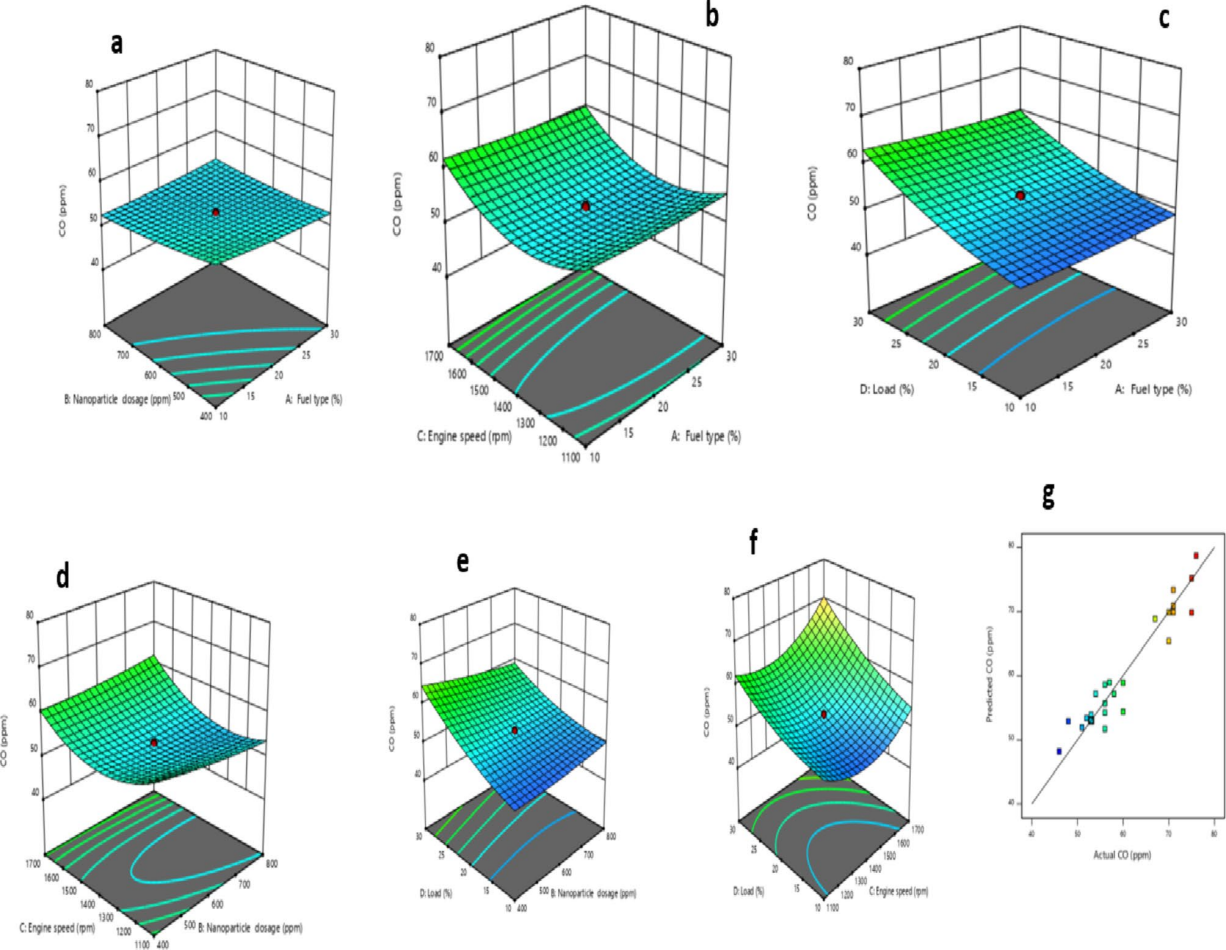
3D based plot for CO: (**a**) Fuel type and nanoparticle; (**b**) fuel type and engine speed; (**c**) fuel type and load, (**d**) nanoparticle dosage and engine speed, (**e**) nanoparticle dosage and load, (**f**) engine speed and load, and (**g**) predicted CO vs. actual CO.

The response surface plots of the CO model are shown in Fig. 16 (a-f), and the variation of RSM predicted CO and actual CO is depicted in Fig. 16g. Figure 16a shows 3D representations of CO emissions compared to fuel type and nanoparticle dosage. As observed, as the fraction of biodiesel in fuel types and dosage of nanoparticle increased, there is a reduction in CO emission. Lower CO emissions could be due to the oxygen in biofuels, which result in easier burning at higher temperatures in the cylinders^94,95^. In addition, high nanoparticle dosage in biodiesel blends was another contributing factor to lower reduction of CO emissions of NCSOME proportion.

Figure 16b demonstrates the interaction of fuel types and engine speed vs. CO emission. As noticed, there is a reduction in CO below an engine speed of 1400 rpm. Figure 16c displays the relationship between fuel type, load, and CO emissions. CO levels increased as load exceeded 20%. Figure 16d demonstrates the influence of engine speed and nanoparticle dosage on CO emissions. Increasing nanoparticle dosage led to a decrease in CO emissions. As portrayed in Fig. 16e, more CO emission is observed at higher load with negligible impact of an increase in the dosage of nanoparticle. Figure 16f presents the engine speed and load vs. CO emission. There is higher CO emission beyond an engine speed of 1400 rpm. Papagiannakis et al.^96^ indicated that the increase of liquid fuel supplementary ratio, which is accompanied by a reduction of the total relative air–fuel ratio, favours the CO formation mechanism. Additionally, Fig. 16g displays the correlation between RSM predicted CO and experimental/actual value of CO.

#### NOx model

Table 12 summarises shows that only nanoparticle dosage, engine speed, and quadratic of engine speed terms are significant for model NO_x_ as indicated by the p-value of regression coefficients. Equation (5b) shows the reduced quadratic model for NO_x_.

**Fig. 17:**
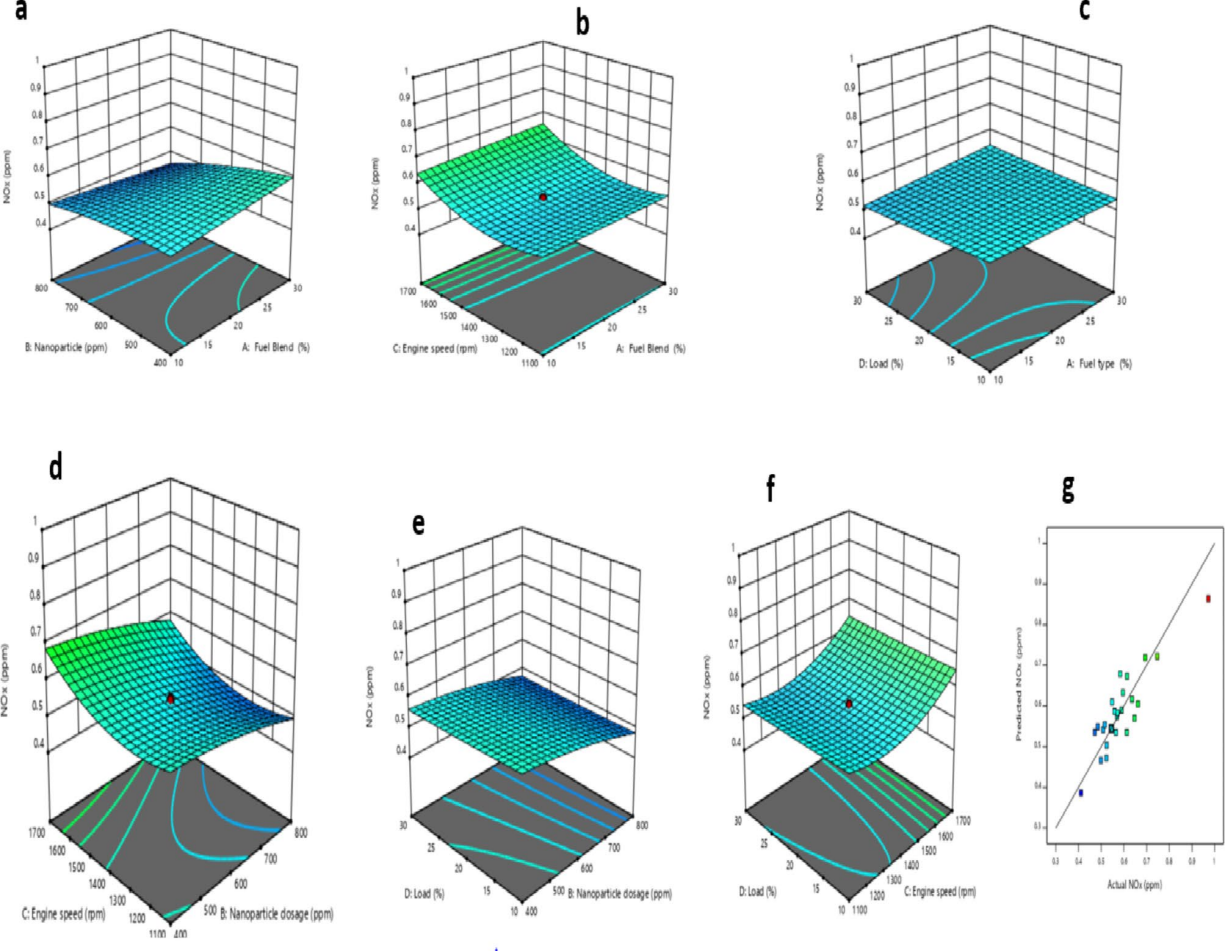
3D based plot for NOx: (**a**) Fuel type and nanoparticle; (**b**) fuel type and engine speed; (**c**) fuel type and load, (**d**) nanoparticle dosage and engine speed, (**e**) nanoparticle dosage and load, (**f**) engine speed and load, and (**g**) predicted NO_x_ vs. actual NO_x_.

The response surface plots of the NOx model are shown in Fig. 17 (a-f), and the variation of RSM predicted NO_x_ and actual NO_x_ is depicted in Fig. 17g. Figure 17a shows 3D representations of NO_x_ emissions compared to fuel type and nanoparticle dosage. As presented, as NCSOME increased in the fuel types, NO_x_ emission increased, while as the dosage of nanoparticles exceeded 600 ppm, there was a reduction in the NO_x_ emission. Similar observations were reported elsewhere^97,98^. Jayaraman et al.^99^ and Khan et al.^65^ attributed the reduction of NO_x_ formation to the synergistic effect of the catalytic property of nanoparticles and heterogeneous combustion leading to lowering the temperature of the cylinder. Figure 17b portrays the interaction between fuel types and engine speed vs. NO_x_. As noticed, as the engine speed increased from 1100 to 1400 rpm, there was a reduction in NO_x_ emission. Beyond the engine speed of 1400 rpm, higher NOx emission was observed. Previous investigations conducted by Wei et al.^100^ and Appavu et al.^101^ revealed an analogous trend. Abdalla et al.^102^ attributed the formation of NO_x_ emission at higher load to the intake of the maximum charge mixture into the cylinder per unit cycle.

The influence of fuel type and load versus NO_x_ was reported in Fig. 17c. As demonstrated, there was no marginal influence with the variations of fuel types and load on NO_x_. The impact of the nanoparticle dosage and engine speed on NO_x_ of IC engine in Fig. 17d. As shown, as nanoparticle dosage increased from 400 to 600 ppm, there was a reduction in the NO_x_, while higher NO_x_ was observed with the increase in the engine speed.

Figure 17e shows the interaction between the nanoparticle dosage and engine load versus NO_x_. As the load and dosage of nanoparticles increased, there was negligible change in the magnitude of NO_x_ emission. However, as the dosage of nanoparticles exceeded 600 ppm, there was a decline in the emission of NO_x_. Figure 17f depicts the interaction of the load and engine speed on the NO_x_ of IC engine. As shown, as the engine speed increased between 1100 and 1400 rpm, there was a reduction in NO_x_ emission. As the speed exceeded 1400 rpm, higher NO_x_ was observed.

#### UHC model

Table 12 shows that only the quadratic terms of the fuel type, nanoparticle dosage, and engine speed are significant for the UHC model, as indicated by the p-values of regression coefficients. Equation (5c) represents the reduced quadratic model for UHC.

**Fig. 18:**
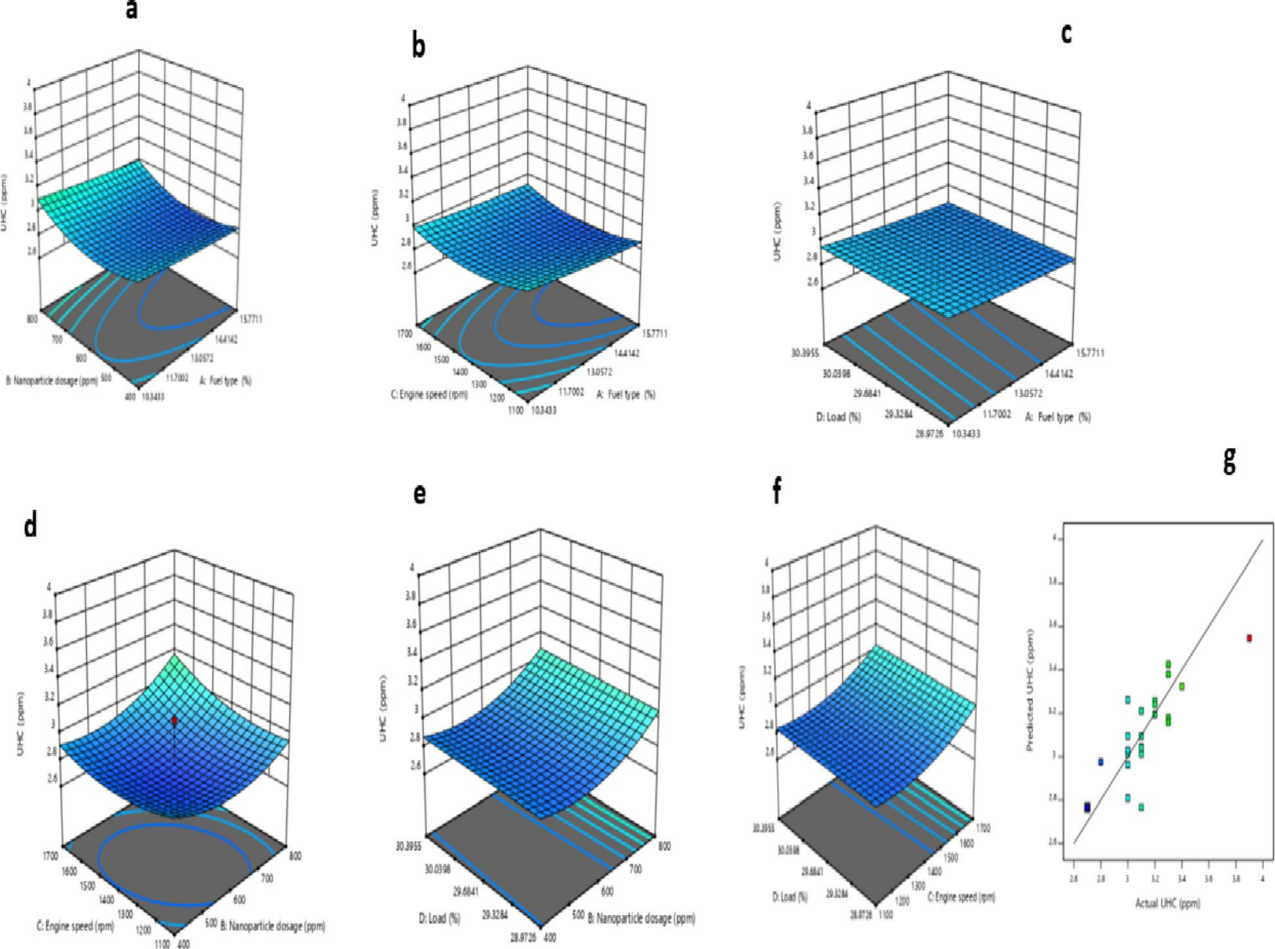
3D based plot for UHC: (**a**) Fuel type and nanoparticle; (**b**) fuel type and engine speed; (**c**) fuel type and load, (**d**) nanoparticle dosage and engine speed, (**e**) nanoparticle dosage and load, (**f**) engine speed and load, and (**g**) predicted UHC vs. actual UHC.

The response surface plots of the UHC model are shown in Fig. 18 (a-f), and the variation between RSM predicted UHC and actual UHC is depicted in Fig. 18g. Figure 18a displays 3D representations of UHC emissions in relation to fuel type and nanoparticle dosage. It is observed that as the fraction of biodiesel and nanoparticle dosage increased, UHC decreased. Similar observations were reported by Devarajan et al.^103^ and Srinivasan et al.^104^. Beyond 20% biodiesel content in the fuel types and 600 ppm nanoparticle dosage, there was a higher UHC emission. This significant increase in unburnt hydrocarbon emissions can be attributed to the non-improved burning of fuel due to the large non-reactive surface of nanoparticles and the oxygenated nature of biodiesel blends^105,106^.

Figure 18b illustrates the impact of fuel type and engine speed on UHC emissions. As the fraction of biodiesel in the fuel type and the engine speed increased, UHC emissions reduced. This finding was also supported by Tormos et al.^107^, Gad et al.^108^, and Chen et al.^109^. Ağbulut et al.^110^ attributed the reduction in UHC emissions at high speed and nanoparticle dosage to the increased oxygenated nature of biodiesel blends. Figure 18c shows the UHC of a ternary fueled engine vs. fuel types and load. The UHC marginally increased with the increase in biodiesel blends, but there was no evidence of variation in UHC with the variation in load. Similar observations were reported by Killol et al.^111^.

Figure 18d presents the UHC of a ternary fueled engine vs. nanoparticle dosage with engine speed.

An increase in engine speed from 1100 to 1600 rpm and nanoparticle levels from 600 to 800 ppm causes an increase in UHC emissions. The emission of UHC increased as the engine speed and nanoparticle dosage increased from1100 to1600 rpm and 600 to 800 ppm, respectively. An increase in engine speed and nanoparticle level causes an increase in unburnt hydrocarbon emissions.

Figure 18e displays the interaction of load and level of nanoparticle (LON) against UHC. The UHC decreased as the LON increased from 400 to 600 rpm. However, the UHC emission increased as the LON exceeded 600 rpm, while there was no significant change in UHC with the variation in load. These results were consistent with the findings of Atarod et al.^112^. The primary causes of this increase in the quantity of UHC emissions could be attributed to unimproved combustion characteristics and inadequate catalyst activity^96^. The influence of load and engine speed (ES) on UHC is shown in Fig. 18f. UHC increased as the load and ES increased. A similar increasing trend of UHC with the increase in the and ES was reported by Glewen et al.^113^ and Singh et al.^114^. Higher load and speed worsen HC emissions by retarding combustion, thus preventing complete combustion^115^.

#### Statistical indices for emission models

Table 13 provides a summary of the RSM model for emission features of NCSOME/diesel operated on an IC engine. As observed, the RSM models are consistent with the literature^116,117^. The inconsistency in this model with existing studies in the literature can be linked to the emission features circumstances, and engine input variables. The accuracy of the RSM model is further validated by the higher values of R^2^ and lower values of other statistical indices such as MAE and AAD compared to the RSM model.

**Table 13:** Emission feature models using statistical indices (SI) and comprised with existing literature.

Area of application	SI for the emission model of Palm oil-diesel blend	SI for the emission Turkish biodiesel-diesel blend	SI for the emission model for NCSOME-diesel blends
R^2^			
UHC	0.9055	0.837	0.7272
CO	0.9375	0.782	0.9234
NO_x_	0.9238	0.984	0.7833
MAE/MRE			
UHC	0.4476	9.98%	1.67E–3
CO	0.7894	10.12%	2.33E–3
NO_x_	0.6438	6.82%	9.252E–18
AAD			
UHC	NA	NA	1.667E–1
CO	NA	NA	2.33E–1
NO_x_	NA	NA	9.252E–16
Refs.	Uslu^116^	Aydın et al.^117^	Present study

#### Optimization and validation of engine operating variables (EOVs) for a green environment

The goal of setting EOVs is to ensure a cleaner environment, which can be achieved within the limitations for the best outcomes. To reach this target, a desirable technique was adopted to optimize the EOVs by minimizing environmental features (EFs) such as CO, NO_x_, and UHC.

**Fig. 19:**
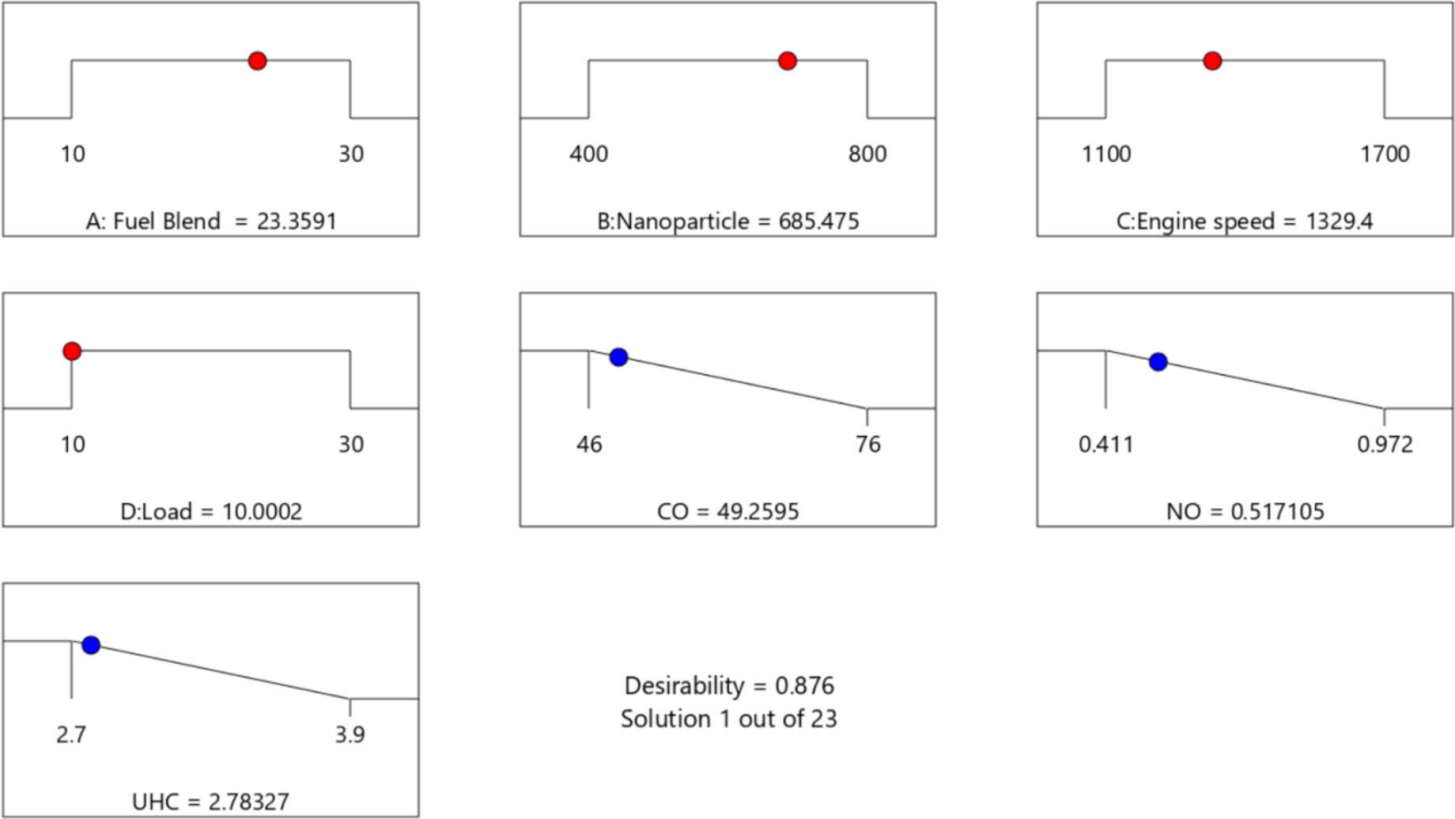
EOVs for a green environment and its optimal solution.

Figure 19 presents optimal solutions indicating the desirability of EOVs and EFs adopted in the investigation. A combined desirability of 0.87 was selected for the optimization. The optimized values were NCSOME in the fuel type at 23.4%, nanoparticle (ZnO) dosage at 685.43 ppm, engine speed at 1329.4 rpm, and load at 10%. The model predicted EFs at the optimized conditions were: 49.26 ppm of CO, 0.52 ppm of NOx, and 2.78 ppm of UHC. Garcia Tobar et al.^118^ observed similar lower values for their NO_x_ (0 ppm) and CO emissions (1.2%) for oil powered emission tests using the Brain Bee AGS-688 analyzer. The reduction in NOx and CO emissions is due to the high dosage of nanoparticles, which enhances the oxidation stability of biodiesel, prevents oxidation, and lowers NOx and CO emissions^42,119^.

In addition to optimisation, a validation assessment is required to check the accuracy of the optimal conditions. Figure 19 present the optimal condition for the emission features for NCSOME/diesel/nanoparticle operated on an IC engine. As shown, the CO (49.26 ppm), NOx (0.5171 ppm), UHC (2.783 ppm) was optimum at fuel blend of 23.4%, ZnO nanoparticle of 685.6 ppm, engine speed of 1329.4 rpm, load of 10%. The justification test using the optimized experimental factors produced CO (47.64), NOx (0.4972), and UHC (2.693). The average error between the predicted and the actual values was found to be 0.034%, 0.041%, and 0.033% for CO, NOx, and UHC, respectively (See Table 14). The validation results showed that the model developed was accurate since the percentages of error in prediction were in good agreement.

**Table 14:** Validation results.

NCSOME content in the fuel type (%)	Nanoparticle (ZnO) dosage (ppm)	Engine speed (rpm)	Load (%)	Value	CO (ppm)	NO_x_ (ppm)	UHC (ppm)
23.4	685.55	1329.4	10.0	Optimized	49.26	0.5171	2.783
				Actual	47.64	0.4972	2.693
				Error (%)	0.034	0.049	0.033

## Conclusion

The study introduced a new method for producing composite biodiesel and predicting kinematic viscosity (KV) using a blend of neem and castor oil (NCSO) with a heterogeneous catalyst (HC) made from egg shell and cow bone. The catalyzed process was conducted on a batch platform using Artificial Neural Network (ANN) and Adaptive Neuro-Fuzzy Inference System (ANFIS) techniques. Models were developed to assess emission features and environmental friendliness indices (EFI) like carbon monoxide (CO), oxides of nitrogen (NOx), and unburnt hydrocarbon (UHC) based on varying engine inputs such as fuel types/NCSOME-diesel (10–30 vol%), ZnO nanoparticle dosage (400–800 ppm), engine speed (1100–1700 rpm), and engine load (10–30%).

Future research recommendations include studying the kinetics of HC, investigating performance and combustion characteristics, establishing a flow diagram for nanoparticle dispersion with NCSOME and diesel, and examining performance and emissions at higher load conditions (75–80%). Future studies should further adopt cross-validation methods, such as k-fold validation as this approach tends to provide greater reliability. The study’s key conclusions highlight the potential for composite biodiesel production and emission reduction in engine operations.

- The ANN architecture exhibits four transesterification parameters, twelve hidden layers, and two output layers in predicting the yield and KV of NCSOME.
- The ANFIS displays 55 nodes, 80 linear parameters, 24 non-linear parameters, 16 fuzzy rules, and 16 inference rules for each input, which were sufficient to represent process performance.
- The ANFIS model showed superior performance in terms of R, R^2^, RMSE, SEP, MAE, and AAD compared to the ANN model in predicting the yield and KV for NCSOME.
- The commercial viability of the novel NCSOME is established since the major fuel properties comply with global biodiesel standards and are consistent with the literature.
- Quadratic equations were established for minimizing exhaust emissions of novel CNSOME-diesel doped with ZnO operated in an IC engine.
- The suitability of RSM for predicting the emission profile is evident as the statistical metrics show a good regression coefficient (R^2^) and lower MAE and AAD.

## Electronic supplementary material

Below is the link to the electronic supplementary material.

Supplementary Material 1

## Data Availability

The data is available within the manuscript.

## Abbreviations

AOA: Arithmetic optimization algorithm
ANN: Artificial Neural Network
C_d_: Catalyst dosage
CCD: Central composite design
CV: Cetane number
DFA: Dragon fly algorithm
HEC: Heterogeneous catalyst
HB: Hybrid biodiesel
KV: Kinematic viscosity
M/NCSO: Methanol to neem-castor oil molar ratio
MPS: Modified particle swarm
NCSOME: Neem-castor oil methyl ester
n: Number of data
R_te_: Reaction temperature
R_t_: Reaction time
UHC: Unburnt hydrocarbon
Z_i,p_: Predicted value
Z_i,e_: Experimental value
Z_e,ave_: Average experimental value
