# Supplementary material for: ANN-ANFIS model for optimising methylic composite biodiesel from neem and castor oil and predicting emissions of the biodiesel blend
Source: Sci Rep. 2025 Feb 15;15:5638. doi: 10.1038/s41598-025-88901-9 (PMC11830028; doi:10.1038/s41598-025-88901-9)
Supplement: Supplementary file 1 — Supplementary Material 1 [file 41598_2025_88901_MOESM1_ESM.docx]

**Supplemenary/Appendices**

**Appendix A**

Table AI: Matrix for the emission of ternary fuel of neem-castor/diesel doped with Zno nanoparticle blend

|  | **Factor 1** | **Factor 2** | **Factor 3** | **Factor 4** | **Response 1** | **Response 2** | **Response 3** |
| --- | --- | --- | --- | --- | --- | --- | --- |
| Runs | A: Fuel Blend | B:Nanoparticle | C:Engine speed | D:Load | CO | NO | UHC |
|  | % | ppm | rpm | % | ppm | ppm | ppm |
| 1 | 10 | 400 | 1100 | 10 | 56 | 0.59 | 3 |
| 2 | 30 | 400 | 1100 | 10 | 53 | 0.636 | 3 |
| 3 | 10 | 800 | 1100 | 10 | 51 | 0.509 | 3.2 |
| 4 | 30 | 800 | 1100 | 10 | 60 | 0.499 | 3 |
| 5 | 10 | 400 | 1700 | 10 | 56 | 0.614 | 3 |
| 6 | 30 | 400 | 1700 | 10 | 56 | 0.694 | 3 |
| 7 | 10 | 800 | 1700 | 10 | 58 | 0.548 | 3.2 |
| 8 | 30 | 800 | 1700 | 10 | 57 | 0.516 | 3.1 |
| 9 | 10 | 400 | 1100 | 30 | 71 | 0.565 | 3.1 |
| 10 | 30 | 400 | 1100 | 30 | 70 | 0.662 | 3.1 |
| 11 | 10 | 800 | 1100 | 30 | 60 | 0.524 | 3.3 |
| 12 | 30 | 800 | 1100 | 30 | 56 | 0.523 | 3 |
| 13 | 10 | 400 | 1700 | 30 | 75 | 0.596 | 3.1 |
| 14 | 30 | 400 | 1700 | 30 | 70 | 0.747 | 3.1 |
| 15 | 10 | 800 | 1700 | 30 | 71 | 0.559 | 3.3 |
| 16 | 30 | 800 | 1700 | 30 | 75 | 0.57 | 3.9 |
| 17 | 0 | 600 | 1400 | 20 | 56 | 0.613 | 3.4 |
| 18 | 40 | 600 | 1400 | 20 | 48 | 0.485 | 3.2 |
| 19 | 20 | 200 | 1400 | 20 | 54 | 0.572 | 3.3 |
| 20 | 20 | 1000 | 1400 | 20 | 52 | 0.411 | 3.3 |
| 21 | 20 | 600 | 800 | 20 | 67 | 0.584 | 3.1 |
| 22 | 20 | 600 | 2000 | 20 | 76 | 0.972 | 3.2 |
| 23 | 20 | 600 | 1400 | 0 | 46 | 0.647 | 3 |
| 24 | 20 | 600 | 1400 | 40 | 71 | 0.472 | 2.8 |
| 25 | 20 | 600 | 1400 | 20 | 53 | 0.545 | 3.1 |
| 26 | 20 | 600 | 1400 | 20 | 53 | 0.545 | 2.7 |
| 27 | 20 | 600 | 1400 | 20 | 53 | 0.545 | 2.7 |
| 28 | 20 | 600 | 1400 | 20 | 53 | 0.545 | 2.7 |
| 29 | 20 | 600 | 1400 | 20 | 53 | 0.545 | 2.7 |
| 30 | 20 | 600 | 1400 | 20 | 53 | 0.545 | 2.7 |

**Appendix B**

Table B1. Physicochemical properties of castor and neem seed oil

| **Types of oils** | **AV**  **(mgKOH/g)** | **SV**  **(mgKOH/g)** | **Density (kg/ m^3^)** | **KV**  **(mm^2^/s)** | **Refs.** |
| --- | --- | --- | --- | --- | --- |
| CSO100 | 12.643 | 207.97 | 0.9477 | 89.784 | Addendum to PS |
| ^*^NSO100 | 18.135 | 201.40 | 0.9082 | 48.787 | Addendum to PS |
| N20CS80 | 14.274 | 202.39 | 0.9361 | 71.562 | PS |
| N100 (NSO) | 3.08 | NA | 0.9040 | 8.83 | Agu et al. [26] |
| RSO50F050 | 22.4 | 193 | 0.948 | 32 | Srikanth et al. [60] |
| CI20CP80 | 17.27 |  | 0.9049 | 26.96 | Ong et al. [61] |

PS=Present study; CI20CP80= Calophyllum inophyllum-Ceiba pentandra oil mixture (20:80 wt%);

N20CS80 =Neem –castor seed oil (20:80 wt%); RSO50F050 = rubber seed-fish oil mix (50:50 wt%)

Table B2. Comprehensive details of equipment and related international standard employed for physicochemical properties of castor and neem seed oil mix

| **Properties** | **Standard method** | **Castor seed oil (CSO)** | **Neem seed oil (NSO)** | **10% NSO**  **+90%CSO** | **20% NSO**  **+80%CSO** | **30% NSO**  **+70%CSO** | **40% NSO**  **+60%CSO** | **50% NSO**  **+50%CSO** |
| --- | --- | --- | --- | --- | --- | --- | --- | --- |
| Nomenclature in the RSM for Esterification | ….. |  |  | 10 | 20 | 30 | 40 | 50 |
| Acid value, (AV) (mgKOH/g) | ASTM D664 | 12.643 | 18.135 | 13.12 | 14.274 | 15.387 | 14.135 | 15.577 |
| Saponification value (mgKOH/g) (SV) | ASTM D94 | 207.97 | 201.40 | 205.56 | 202.39 | 205.71 | 205.95 | 206.52 |
| Density (kg/ m^3^ | ASTM D1250 | 0.9477 | 0.9082 | 0.9353 | 0.9361 | 0.9347 | 0.9295 | 0.9282 |
| Kinematic viscosity (mm^2^/s) | ASTM D445 | 89.784 | 48.787 | 79.837 | 71.562 | 64.604 | 61.132 | 57.428 |
| Free fatty acid (%) | ASTM D974 | 6.322 | 9.067 | 6.56 | 7.137 | 7.693 | 7.067 | 7.788 |
| Moisture  Content (%) | ASTM D95 | 0.690 | 0.563 | 0.678 | 0.6221 | 0.6247 | 0.625 | 0.626 |
| Molecular weight | ……. | 861.632 | 918.342 | 874.558 | 894.661 | 884.286 | 877.41 | 881.415 |
